# First-Principles Models of Polymorphism of Pharmaceuticals: Maximizing the Accuracy-to-Cost Ratio

**DOI:** 10.1021/acs.jctc.4c00099

**Published:** 2024-03-26

**Authors:** Jan Ludík, Veronika Kostková, Štefan Kocian, Petr Touš, Vojtěch Štejfa, Ctirad Červinka

**Affiliations:** Department of Physical Chemistry, University of Chemistry and Technology Prague, Technická 5, CZ-166 28 Prague 6, Czech Republic

## Abstract

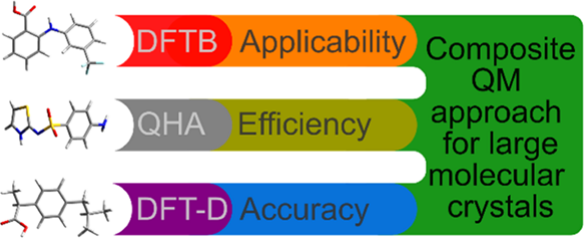

Accuracy and sophistication of in silico models of structure, internal dynamics, and cohesion of molecular materials at finite temperatures increase over time. Applicability limits of ab initio polymorph ranking that would be feasible at reasonable costs currently represent crystals of moderately sized molecules (less than 20 nonhydrogen atoms) and simple unit cells (containing rather only one symmetry-irreducible molecule). Extending the applicability range of the underlying first-principles methods to larger systems with a real-life significance, and enabling to perform such computations in a high-throughput regime represent additional challenges to be tackled in computational chemistry. This work presents a novel composite method that combines the computational efficiency of density-functional tight-binding (DFTB) methods with the accuracy of density-functional theory (DFT). Being rooted in the quasi-harmonic approximation, it uses a cheap method to perform all of the costly scans of how static and dynamic characteristics of the crystal vary with respect to its volume. Such data are subsequently corrected to agree with a higher-level model, which must be evaluated only at a single volume of the crystal. It thus enables predictions of structural, cohesive, and thermodynamic properties of complex molecular materials, such as pharmaceuticals or organic semiconductors, at a fraction of the original computational cost. As the composite model retains the solid physical background, it suffers from a minimum accuracy deterioration compared to the full treatment with the costly approach. The novel methodology is demonstrated to provide consistent results for the structural and thermodynamic properties of real-life molecular crystals and their polymorph ranking.

## 1 Introduction

Progress of the computational methodologies over the last two decades enabled to achieve impressive predictions of structural and thermodynamic properties of model molecular crystals, including their densities,^1−4^ anisotropy,^5,6^ phonon spectra,^7−9^ lattice energies,^10,11^ and finite-temperature thermodynamic properties,^8,12−14^ in particular sublimation enthalpies,^15,16^ sublimation pressures,^17,18^ and ultimately, polymorph ranking at finite temperatures and pressures.^6,7,19,20^ Computational complexity of predicting individual properties increases in a row: lattice energy < sublimation enthalpy ≈ enthalpic polymorph ranking < sublimation pressure ≈ solid–solid phase-transition temperatures, with the finite-temperature contributions to enthalpy and free energy being substantial to achieve a proper ranking.^19^

Several relatively broad benchmark sets (C21,^21^ X23,^15,22^ Z20,^16,17^ G60,^23^ PV17,^24^ and other^25^) of small-molecular crystals have been devised to assess the accuracy of newly developed first-principles models of crystal cohesion. While reaching the chemical accuracy (≈4 kJ mol^–1^) for predictions of the enthalpic data has become standard for contemporary methods,^26^ more modest milestones are still typically targeted for the remaining equilibrium properties, which are based on the equality of Gibbs free energies of coexisting phases. Capturing the correct order of magnitude of the sublimation pressures^17^ or approaching the actual temperature of an enantiotropic phase transition to within a few dozens of degrees Kelvin still represents the current state-of-the-art.^6,19^ To achieve such goals, a subkilojoule-per-mole accuracy on the energetic scale has to be targeted.^27^

All of the abovementioned studies had one aspect in common that is focusing primarily on reaching a high computational accuracy and on exploiting rather complex theories, often requiring considerable computational resources. For this reason, all studies covered mostly crystals of small model molecules, containing less than 10–15 nonhydrogen atoms per molecule. Nevertheless, the main interest in the development of first-principles models for molecular crystals is now focused on active pharmaceutical ingredients (API) or organic semiconductors (OSC), not forgetting, however, also other applications in areas of food processing, dyes, explosives, fertilizers, etc. Molecules of such materials possessing a real significance are frequently significantly larger, containing typically dozens of nonhydrogen atoms, imposing significant computational requirements in cases of real API^28^ and OSC.^29,30^ Modeling properties of such materials at finite temperatures from first principles remains challenging due to the computational cost,^31^ related numerous conformational degrees of freedom,^27^ or possible occurrence of local disorder.^32^ Development of novel methods, enabling high-throughput in silico ranking of candidate structures, is also highly desirable from the perspective of crystal structure prediction.^33^

Therefore, within the development of new computational protocols, it has become very desirable to focus not only on improving the accuracy but also on the aspect of extending the applicability range of the underlying models to crystals of larger molecules with a real significance. There have been initiatives to reduce the prohibitive cost of phonon calculations using machine-learned potentials,^34^ fragment-based approaches,^9^ or employing semiempiric quantum-chemical methods for the expensive parts of the computational protocols.^35^

Novel development achieved within this manuscript also fares in the latter direction, aiming at combining the established quasi-harmonic density-functional theory (DFT) protocols with efficient semiempiric density-functional tight-binding (DFTB) approaches. Replacing the most expensive DFT tasks with cheaper DFTB alternatives in a way that would not harm the original accuracy of the full DFT model would enable performing first-principles predictions for a significantly broader scope of molecules, thus being highly desirable in numerous areas of material research. We demonstrate that performing costly DFT calculations for a single-crystal volume and treating the variation of both the static and dynamic characteristics with respect to volume with DFTB proves to be an excellent approach to concurrently retain the original high accuracy of DFT quasi-harmonic models and to save the majority of initial computational requirements of a corresponding full DFT model.

**Figure 1:**
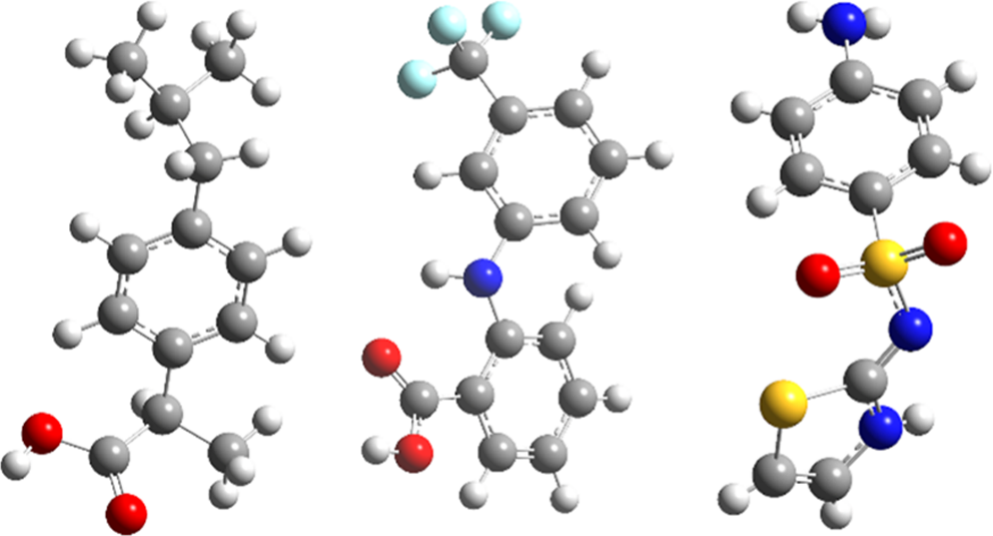
Molecular structures of target active pharmaceutical ingredients: left, ibuprofen; center, flufenamic acid; right, sulfathiazole.

Seven crystal structures of three archetypal API, namely, ibuprofen (both racemic and enantiopure), flufenamic acid, and sulfathiazole (depicted in Figure 1), all known to exhibit polymorphism, are selected to demonstrate the improved efficiency of a composite quasi-harmonic first-principles model. Unit cells of the crystal structures considered in this work are depicted in Figure S1.

**Figure 2:**
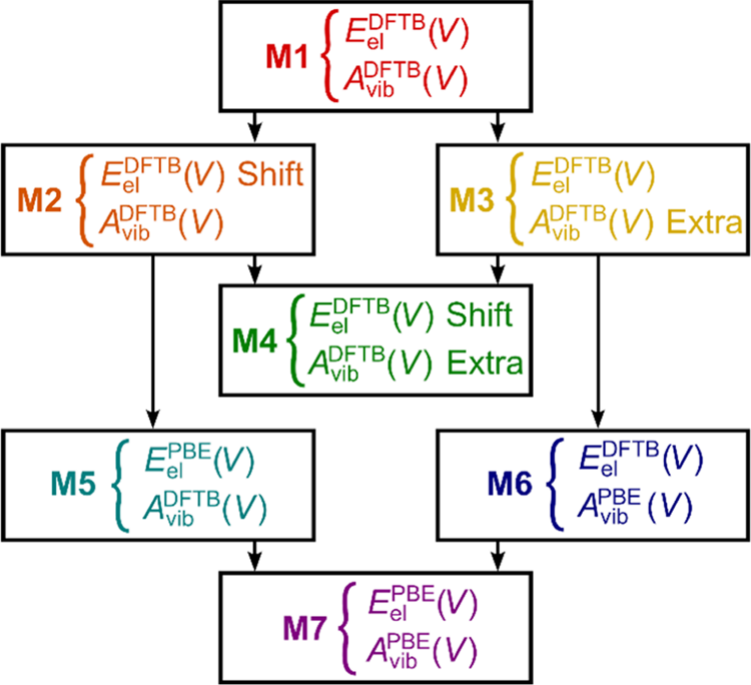
Relationships of individual composite quasi-harmonic approaches, using either DFTB3-D4/3ob or PBE-D3(BJ)/PAW levels of theory for the description of the static electronic energies *E*_el_(*V*) and vibrational Helmholtz energies *A*_vib_(*V*) of the crystals. Vertical arrows indicate replacing one of these components with a more sophisticated or more costly approach.

Full DFT (with the PBE functional) and full DFTB methodologies stand at the opposite poles of the spectrum of the computational protocols in this work: a relatively accurate but expensive method against another cheap but not that accurate method. The quasi-harmonic approximation (QHA)^8,36^ includes two principal components, dealing either with static electronic energy of the crystal or with its dynamic characteristics (phonons). Following the QHA logic, its individual components, obtained using either the DFT or the DFTB levels of theory, are combined here in multiple ways to yield five composite computational protocols. Relationships thereof are illustrated in Figure 2. Literature data on crystal densities and new calorimetric determinations serve as sources of reliable reference data for the validation of the newly presented computational protocols.

## 2 Methodology

All initial crystal structures of the studied compounds were taken from the Cambridge Structural Database (CSD).^37^ Crystal structures primarily investigated in this work are listed in Table 1, whereas more information on the unit-cell parameters is given in Table S1. Note that molecules containing 15–20 nonhydrogen atoms were selected as targets in this work so that our computational models could be tested directly for real API materials. Although there are definitely significantly larger API molecules in the market, such constraints on the molecular size enabled us to retain the computational cost of our benchmark within an affordable range. The most stable form of flufenamic acid, FFA_3_, was excluded from our benchmark due to the significant computational requirements associated with modeling its large unit-cell. Individual polymorph pairs considered for each target API are relatively similar in terms of the space group and crystal packing motif, mostly differing in the monoclinic tilt, mutual orientations of neighboring molecules, or extent of their anisotropy. This structural similarity may somewhat limit the statistical width of the accuracy assessment of our computational models, but it appreciably emphasizes the challenges associated with the polymorph stability ranking. Our target crystal structures vary to some extent in terms of the anisotropy of the noncovalent cohesive interactions, covering both rather weakly anisotropic structures where hydrogen bonding manifests along all crystal-lattice vectors (sulfathiazole) and moderately anisotropic structures with more direction-specific hydrogen bonding (flufenamic acid). Still, our test set does not include any strongly anisotropic structure that would be held of parallel planar sheets that are hydrogen-bonded inside, but relying only on dispersion interactions in the perpendicular direction. Identification of the most important interaction types along individual lattice vectors is listed in Table S2.

**Table 1:** Overview of the Compounds and Their Crystal Structures Primarily Considered in This Work

trivial name	formula	structure	CSD refcode	*Z*	space group	*T*_fus_/K
ibuprofen	C_13_H_18_O_2_	IBU_RS1_	IBPRAC16^38^	4	*P*2_1_/*c*	348.4^39^
		IBU_RS2_	IBPRAC04^40^	4	*P*2_1_/*c*	291.0^39^
		IBU_S_	JEKNOC12^41^	4	*P*2_1_	324.4^39^
flufenamic acid	C_14_H_10_F_3_NO_2_	FFA_1_	FPAMCA17^42^	4	*P*2_1_/*c*	407.7^42^
		FFA_2_	FPAMCA18^43^	4	*P*2_1_/*c*	402.8^42^
sulfathiazole	C_9_H_9_N_3_O_2_S_2_	STZ_3_	SUTHAZ35^44^	8	*P*2_1_/*c*	n/a^a^
		STZ_4_	SUTHAZ45^44^	4	P2_1_/*n*	n/a

a Melting was observed only for STZ_1_ at 475 K and for STZ_5_.^45^

### 2.1 Periodic DFT Calculations

Generalized-gradient approximation, namely, the PBE functional,^46^ along with the D3(BJ) model of dispersion interactions^47^ was used as a reference DFT level of theory for all other methods developed in this work. As implemented in VASP, version 5.4.4,^48,49^ the projector-augmented wave formalism (PAW)^50^ with a plane-wave kinetic energy cutoff of 1000 eV and hard PAW potentials^51^ was employed. A Γ-point centered Monkhorst–Pack grid^52^ with roughly 25/*a*_*i*_*k*-points along each reciprocal unit-cell vector (*a*_*i*_ stands for the lattice parameters in Ångstrom) was used to sample the first Brillouin zone. Such a computational setup has been extensively validated previously for various molecular crystals.^3,4,8,53^ It will be termed PBE hereafter. More details on the computational setup are given in Table S3.

### 2.2 Periodic DFTB Calculations

Third-order DFTB3 calculations, including a Hubbard U-parameter augmentation of the self-consistent charge (SCC) Hamiltonian,^54^ were performed in the DFTB+ code, version 21.1.^55^ The 3ob parametrization^56,57^ for organic molecules was employed, significantly reducing errors in overbinding bulk structures and reducing errors in calculated noncovalent interactions. A damping method with an exponent of 4.05 was chosen for the contribution of short-range hydrogen interactions to the SCC.^54^ D4 model^58^ was used to model the dispersion interactions. This approach will be termed DFTB hereafter.

### 2.3 Quasi-Harmonic Processing

Raw DFTB and raw PBE quasi-harmonic protocols,^8,36^ denoted M1 and M7 in Figure 2, respectively, were followed at first separately using an analogous workflow. It consists of two major steps. First, an *E*_el_(*V*) curve, representing the response of the static electronic energy to the variation of crystal volume, is constructed and fitted with the Murnaghan equation of state.^19^ The underlying unit-cell optimizations performed at constrained volumes enable an independent variation of the three lattice vectors (in VASP at the PBE level), mimicking the crystal anisotropy upon variation of its volume. Subsequently, dynamic degrees of freedom of the crystal, that is, its phonon characteristics, are modeled. Phonons were modeled using the finite-displacement method for supercells exceeding at least 10 Å, as implemented in the Phonopy code, version 1.9.^59^ Phonons and *E*_el_(*V*) curves were translated into volume- and temperature-dependent Helmholtz energies of the materials, allowing their complete thermodynamic description. More information on the setup of this protocol is given in the Supporting Information (SI) and in our previous publications, focusing on the validation of purely DFT-D quasi-harmonic models.^4,8,60^

### 2.4 Novel Composite Models

Obviously, the most expensive portions of the computational workflow consist of repeating the geometry optimizations and phonon calculations for different crystal volumes. Our initiative was based on exploiting as much as possible from the cheap DFTB calculations of these volume-dependent trends but correcting their outcomes to match the PBE calculations at a single reference volume. As depicted in Figure 2, our novel improvements of any potential flaws of the M1 approach were first based on correcting either only the static *E*_el_ ingredient (M2), or the dynamic characteristics affecting the *A*_vib_ terms (M3).

The idea behind the M2 model, labeled in Figure 2 as “Shift”, is a horizontal shift of the whole *E*_el_(*V*) curve obtained from cheap DFTB calculations so that its minimum matches that of the *E*_el_(*V*) curve obtained from more costly PBE calculations. A key parameter for this transformation is the horizontal shift Δ*V*_0_, defined as
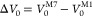
1

The shift of the DFTB *E*_el_(*V*) curve can then be mathematically expressed as
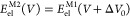
2

To obtain the whole *E*_el_^M2^(*V*) curve, it suffices to perform only one unit-cell optimization at the more costly level of theory. Incorporating the position of the minimum of the M7 *E*_el_(*V*) curve in the M2 model is expected to improve the quality of predicted equilibrium densities. Adopting the *E*_el_(*V*) curve from PBE calculations without any shifts and its combination with raw DFTB phonons yields the model M5.

The idea behind the M3 model is to exploit the volume dependence of phonons obtained from cheap DFTB calculations, extract the Grüneisen parameters therefrom, and use these parameters to extrapolate the volume dependence of the higher-level PBE phonons. Adopting this procedure, the costly PBE phonon calculation can be performed only for a single volume. The fact that the PBE quality of phonons is incorporated in the M3 model raises expectations to beneficially impact zero-point energies and other finite-temperature corrections to the thermodynamic properties of the crystals.

For this purpose, we adopted the microscopic definition of the Grüneisen parameter γ_*i*_ for the *i*th phonon mode, dealing with the derivative of its frequency ν_*i*_ with respect to the crystal volume *V*, as defined in eq 3
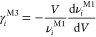
3However, we did not perform the phonon mode matching in terms of the similarity of eigenvectors of individual phonon modes. To emphasize the high-throughput nature of the “Extra” correction, and to avoid significant amounts of labor required to match the eigenvectors of individual modes, we only matched the phonon modes among individual crystal structures (differing in their volume) according to their position in a sorted frequency list. In other words, to establish the Grüneisen parameter for the phonon mode number *N*, we grouped together the *N*th lowest phonon frequencies for each crystal volume and interpolated their volume dependence with an exponential function given in eq 4, resulting straightforwardly from (eq 3)
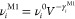
4containing two adjustable parameters ν_*i*_^0^ and γ_*i*_^M1^ for each mode. Finally, the M3 frequencies were accessed as
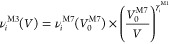
5

Concurrent application of both the modifications that are performed within M2 and M3 models then provides the M4 model, possessing the highest potential to maximize its accuracy-to-cost ratio among all of the considered seven models. Adopting the *E*_el_(*V*) curve from DFTB calculations without any shifts, and its combination with raw PBE phonons yields the model M6. More details on the setup of the composite models are given in Table S4.

### 2.5 Fragment-Based Ab Initio Refinements

To refine the static electronic lattice energies of all ranked polymorphs, a fragment-based protocol was followed.^19^ Within the PBE optimized crystal structures, corresponding to the minimum of an *E*_el_(*V*) curve, all symmetry-unique molecular pairs were identified within a cutoff distance of 8 Å. Within this model, the dimers and respective monomers were placed in a void virtual cubic cell spanning 40 Å to obtain their energies within the PAW framework. To improve the description of the energies of these isolated dimers, two advanced ab initio approaches were adopted. First, the explicitly correlated^61^ dispersion-corrected MP2C-F12/aug-cc-pVDZ method was used as an efficient high level of theory^62^ to correct all pair interactions within the given cutoff. Molpro code, version 2019.1,^63^ was used for all MP2C calculations. To correct the monomer energies, depending on the conformational preferences of individual polymorphs,^27^ MP2/aug-cc-pVDZ single-point calculations were used to retain consistency with the dimer treatment. The ab initio corrected lattice energy *E*_lat_^AbIn^ per unit cell can then be written as
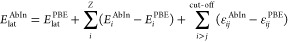
6where the first summation of monomer energies *E*_*i*_ runs over all molecules within a unit cell and the other summation runs of pair interaction energies ε_*ij*_ runs over all pair interactions of molecules *i* and *j* within a cutoff distance.

### 2.6 Calorimetric Experiments

To obtain reliable reference data, flufenamic acid and sulfathiazole samples (description provided in Table S5) were subject to calorimetric investigations of their phase behavior (using heat-flux differential scanning calorimetry)^32^ and determinations of heat capacities (using Tian–Calvet microcalorimetry^64^ and the continual heating method^65^) of selected crystalline phases in the temperature range of 240–350 K. Identity of the treated samples was verified through all of the experimental stages using X-ray powder diffraction. More details on the experimental setup and data curation are provided in Section S2 of the SI.

## 3 Results and Discussion

### 3.1 Reference Data

To enable stringent performance benchmarking of various computational methods, critically assessed reference data are required. To our knowledge, no low-uncertainty thermodynamic data are available in the literature for flufenamic acid and sulfathiazole, although polymorph ranking was performed experimentally for these species.^42,45^ A detailed discussion of the observed phase behavior of flufenamic acid and sulfathiazole over various thermal regimes is given in Table S6 and Figures S2 and S3. Our calorimetric measurements in the temperature range from 240 to 350 K yielded isobaric heat capacity data for multiple targeted polymorphs, the expanded relative uncertainty of which amounts to 0.6% (at the 0.95 level of confidence).^64^ These heat capacity results are presented in detail in Tables S7 and S8 and in Figures S4 and S5. For ibuprofen, a recent thermodynamic study covering also the structures IBU_RS1_ and IBU_S_ was used as the primary source of experimental heat capacity data for the crystalline phase.^39^ Concurrent application of calorimetry and PBE-D3/PAW quasi-harmonic calculations enabled us to derive pseudoexperimental heat capacity and entropy data below 240 K, which are depicted in Figure S6 and listed in Tables S9 and S10, respectively. Reliability of this procedure has been previously tested.^6^

Our novel experiments contribute to disentangling the prevailing controversies on polymorph ranking of flufenamic acid at low temperatures,^42^ confirming that the FFA_3_ polymorph is enantiotropically related to the common FFA_1_ and that FFA_3_ is the stable structure at low temperatures. Data on polymorph ranking in Table 2 suggest that it is very likely that the phases FFA_1_ and FFA_2_ are in a monotropic relationship.

**Table 2:** Experimental Ranking of the Target Polymorphs in Terms of Enthalpy and Gibbs Energy (in kJ mol^–1^) at 300 K and Atmospheric Pressure^a^

compound	transition	Δ*H*	Δ*G*	relationship
ibuprofen	IBU_RS1_ → IBU_RS2_	15.6 ± 1.3^b^	3.7 ± 0.2^b^	monotropic
flufenamic acid	FFA_1_ → FFA_2_	2.2 ± 1.7	0.8 ± 0.6^c^	monotropic
sulfathiazole	STZ_3_ → STZ_4_	0.4 ± 0.5	0.2 ± 0.4^c^	likely monotropic

a Data provided with expanded uncertainties (*k* = 2), which were estimated from the experimental uncertainties of enthalpies and temperatures of phase transitions and heat capacities of all respective phases.

b Based on experimental fusion parameters determined in refs (39,66).

c Based on experimental fusion parameters determined in this work. Data listed in Table S6. Heat capacity difference between the two ranked polymorphs was neglected so that Δ*H* is assumed to be constant and only the phase-transition entropy governs the temperature dependence of Δ*G*. Experimental uncertainty of the heat capacity was still considered to assess the reported uncertainties.

Similarly, our experiments confirm that phase STZ_1_ is the most stable structure at elevated temperatures close to the melting point. Either phase STZ_3_ or STZ_4_ is the stable one under ambient conditions. Experiments suggest that their relationship is monotropic, however, both exhibiting very close energies. Since we did not observe their mutual transformation directly, their ranking could not be definitively established. Profiles of the respective Gibbs energy differences among all observed polymorphs of flufenamic acid and sulfathiazole are displayed in Figures S7 and S8. Since the first observation of ibuprofen polymorphism,^66^ the relationship between IBU_RS1_ and IBU_RS2_ has been established as monotropic, with IBU_RS2_ being the metastable phase.^39^

Structural reference data were gathered from the Cambridge Structural Database,^37^ which contains abundant variable-temperature results for IBU_RS1_, IBU_S_, STZ_3_, and STZ_4_ structures and a sufficient amount of data to derive the thermal expansivity of these materials with a reasonable accuracy. On the contrary, there are only lower units of entries for FFA_1_, FFA_2_, and IBU_RS2_ polymorphs, always related to isolated temperatures, restraining us from deriving reliable thermal expansivity data. Note that the typical interlaboratory scatter of the crystal densities is significant and using only few isolated density points recorded with different experimental setups (sample characteristics, temperature control, etc.) at different temperatures may lead to misleading thermal expansivity data.^6,32^ Isothermal compressibility data could be derived from variable-pressure entries only for IBU_RS1_. More information on experimental density data assessment is given in Figures S9–S12.

### 3.2 Static QHA Components

**Figure 3:**
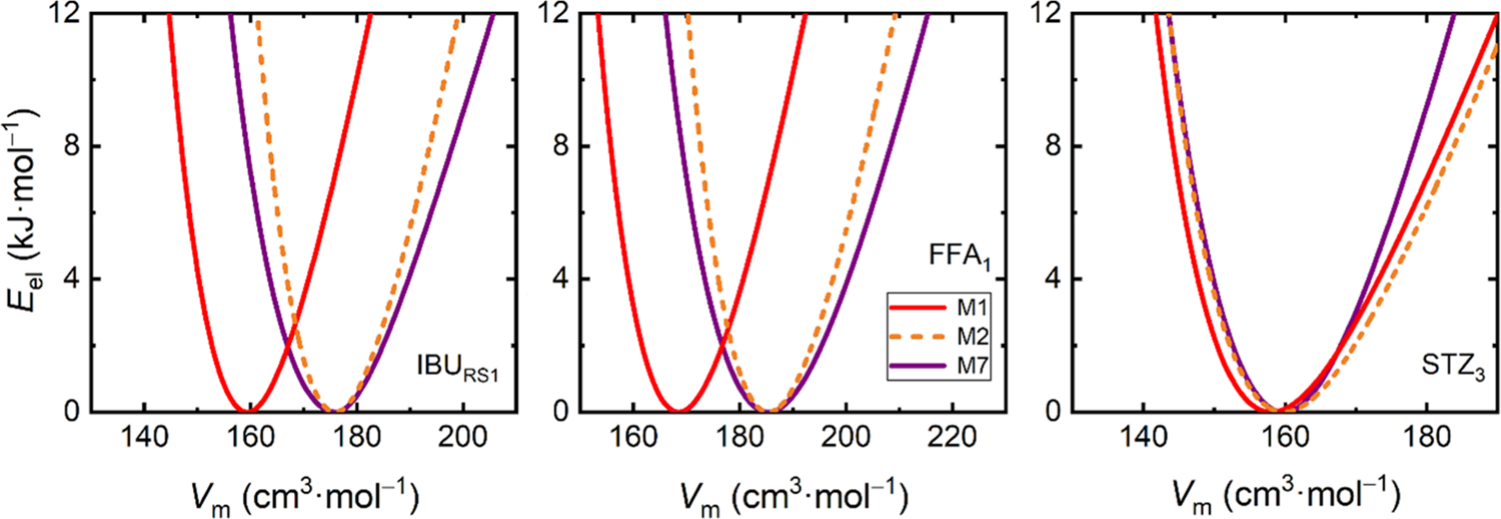
Static electron energy *E*_el_ as a function of the molar volume of the crystal for selected polymorphs of RS-ibuprofen (IBU_RS1_), flufenamic acid (FFA_1_), and sulfathiazole (STZ_3_). Data were calculated using the M1 approach (DFTB3-D4/3ob, red), the M7 approach (PBE-D3(BJ)/PAW, purple), and the M2 approach (M1 curve horizontally shifted to match the M7 minimum).

Variation of the electronic energy with respect to the crystal volume represents the static component of QHA. Corresponding *E*_el_(*V*) curves obtained by PBE and DFTB differed appreciably for most target polymorphs. The most significant feature of this comparison is the horizontal shift of the DFTB curves toward lower volumes when compared to the PBE results, being visible in Figures 3 and S13 for crystalline ibuprofen and flufenamic acid. This volume shift amounts to 8–10% of the equilibrium PBE volume *V*_0_^PBE^ and can be possibly attributed to an overbinding of those crystals at the DFTB level due to basis-set-superposition error. Interestingly, such a shift is only marginal for sulfathiazole, indicating that a fortuitous error cancellation occurs in this case.

Obviously, the position of *V*_0_^PBE^ governs the predicted equilibrium volume of the target material. Merely adopting the *E*_el_(*V*) curve from DFTB can thus propagate to a significant underestimation of the equilibrium volume of the crystal. To avoid such a miscalculation, we propose to perform a horizontal shift of the whole DFTB *E*_el_(*V*) curve so that the position of its minimum *V*_0_^DFTB^ matches the *V*_0_^PBE^ value, as depicted in Figure 3 and Section 2.4. By doing so, the static electronic energy minimum will be fixed at the assumably more realistic *V*_0_^PBE^ value, but its volume dependence will be retained in the shape yielded from the DFTB calculations.

This shift of the whole *E*_el_(*V*) is the idea behind the approach labeled M2 (and M4) in Figure 2. Provided that the underlying PBE/PAW calculations employ a large plane-wave kinetic energy cutoff (as the current 1000 eV), it is sufficient to perform a single such PBE/PAW unit-cell optimization to locate *V*_0_^PBE^, and the description of the whole *E*_el_(*V*) may then rely on the significantly cheaper DFTB calculations, leading to tremendous savings of the computational resources required to generate a single *E*_el_(*V*) curve in the M2 (and M4) approaches. Note that the PBE and DFTB theories yield their *E*_el_(*V*) curves with slightly different curvatures, eventually impacting the predicted thermal expansivity and compressibility of the material. These curvatures differ by about 1/3–2/3 among the DFTB and PBE models as listed in Table S11.

Comparison of experimental unit-cell parameters with their M1 and M7 counterparts, corresponding to the *E*_el_(*V*) minima and optimized in terms of electronic energy only is listed in Table S12. It reveals that the M7-optimized unit-cell parameters are appreciably closer to the experimental values. A closer inspection reveals that DFTB often fails to reproduce the balance between hydrogen bonding and dispersion, which translates into significant prolongation of one of the unit-cell vectors and contraction of another (occurs above all for IBURS2 and STZ3). PBE does not perform well when π–π stacking dominates the cohesion in a given direction, leading to spurious shrinkage of the corresponding lattice vector (occurs for both FFA polymorphs).

### 3.3 Dynamic QHA Components

Vibrational degrees of freedom of all atoms in the crystal lattice, i.e., phonons, and their energies and variation with crystal volume govern the dynamic component of the quasi-harmonic approximation. Thanks to the parametrization strategy of modern semiempiric QM methods, aiming at reproducing also the molecular vibrational frequencies of the stretch modes,^56,67^ current DFTB calculations reproduce higher-frequency C–H stretch modes with a higher accuracy than the systematically overestimated PBE results.^68,69^ Unfortunately, in the thermodynamic context, it is the low-frequency region that governs the macroscopic properties of materials at low-to-ambient temperatures, whereas the high-frequency modes retain their importance for the zero-point energy. Calculated densities of phonon states and identification of the signals of stretching modes affected by hydrogen bonding are given in Figure S14.

**Figure 4:**
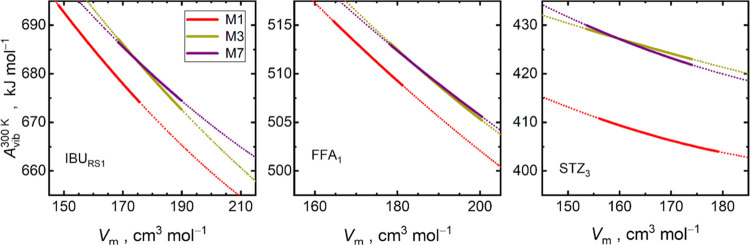
Vibrational Helmholtz energy *A*_vib_ at 300 K as a function of the molar volume for selected polymorphs of RS-ibuprofen (IBU_RS1_), flufenamic acid (FFA_1_), and sulfathiazole (STZ_3_). Data were calculated using either DFTB3-D4/3ob (M1) or PBE-D3(BJ)/PAW (M7) levels of theory and their M3 composite approach extrapolating single-volume PBE-D3(BJ)/PAW frequencies with DFTB3-D4/3ob Grüneisen parameters (M3). Solid lines represent volume intervals covering explicit M1 and M7 phonon calculations, whereas dotted lines represent extrapolations of *A*_vib_(*V*_m_).

Dependence of the phonon densities of states on the crystal volume then propagates into the vibrational contribution to the Helmholtz energy *A*_vib_(*T*, *V*), which are depicted in Figures 4 and S15 and characterized in Table S13. These functions of volume typically exhibit a convex, close-to-linear shape over broad intervals of temperature. *A*_vib_(*V*) functions at 300 K calculated using DFTB and PBE are mutually shifted; however, both exhibit very similar slopes.

Similarity of the slopes of *A*_vib_ curves computed with the raw M1 and M7 methods explains the motivation to formulate the composite M3 approach, aiming at saving the cost of phonon calculations. Phonon dispersion and dependence of phonon frequencies on the crystal volume are cheaply calculated at the DFTB level. The latter can be used to derive Grüneisen parameters,^70^ which can in turn be used to extrapolate the PBE phonon frequencies, explicitly calculated at a single volume (*V*_0_^PBE^) only. Figure S16 confirms a high degree of similarity of the volume dependence of the phonon frequencies obtained from PBE and DFTB. In this way, repeating the most expensive PBE phonon treatment for multiple volumes can be circumvented, and reasonable *A*_vib_(*V*) curves can be obtained with a manifold decrease of the computational costs.

Obviously, the rather crude mode matching adopted for the M3 and M4 models imparts some chaos to the extrapolative correction of phonons. Nevertheless, two facts lead to minimization of any undesirable consequences for this approximation: (i) the fitting with eq 2 is performed over multiple phonon sets covering a broader volume interval that probably averages out any mode mismatches and (ii) evaluated Grüneisen parameters for neighboring modes are highly similar (confirmed in Figure S16).

Since the performance of both the static and dynamic corrections introduced in the M2 and M3 methods can be justified at the microscopic level, their concurrent application resulting in the M4 method is expected to be the logical approach to maximize the accuracy-to-cost ratio of the quasi-harmonic modeling, as will be discussed below.

### 3.4 Predicted State Variables

**Figure 5:**
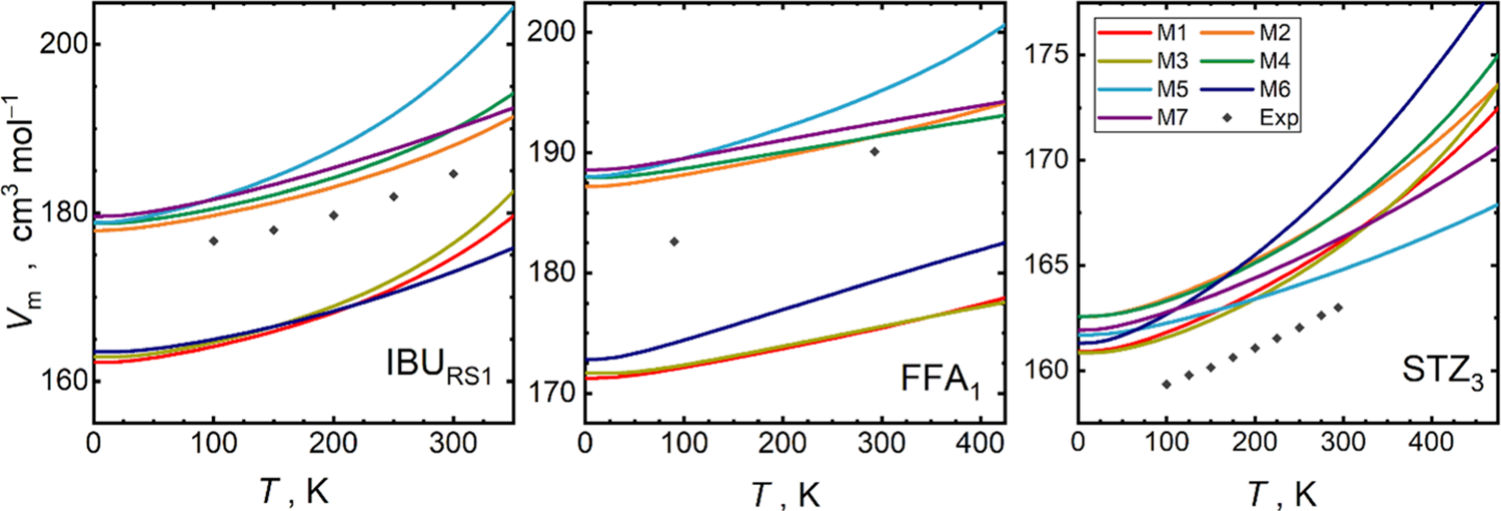
Equilibrium molar volumes for selected target crystal phases at zero pressure calculated using individual formulations of the quasi-harmonic approximation M1–M7 (colored lines, see Figure 2 for explanation), and experimental reference data^38,43,71−74^ (gray diamonds).

State variables such as molar volume (*V*_m_) and absolute molar entropy (*S*_m_) act as the first derivatives of the Gibbs energy with respect to the pressure and temperature, respectively. As such, these properties can be calculated at a fair accuracy level from first principles. Figures 5 and S17 illustrate the trends of quasi-harmonic equilibrium molar volumes for the target materials. M7 results here follow the common pattern of the PBE/PAW performance, generally overestimating *V*_m_ of molecular crystals.^3,4^ For ibuprofen and flufenamic acid, there is a significant discrepancy between the M1 and M7 equilibrium volumes, which differ by 10% (up to 20 cm^3^ mol^–1^) with DFTB always providing lower *V*_m_ results. This overbinding of the crystals by DFTB can be possibly attributed to basis-set superposition issues, not being completely compensated for within the 3ob and D4 parametrizations. This offset of M1 volumes does not occur for sulfathiazole due to the similarity of its PBE and DFTB *E*_el_(*V*) curves.

Comparing the individual formulations of QHA, all approaches sharing the same *E*_el_(*V*) source yield similar molar volumes. Methods M1, M3, and M6, sharing the *E*_el_(*V*) from DFTB, are thus grouped in terms of the resulting *V*_m_, whereas the remaining methods are grouped with a similar offset at a higher *V*_m_. The latter group also ranges closer to the experimental reference data. Calculated *V*_m_ values at 0 and 300 K are tabulated in Table S14.

With respect to the availability of experimental data, we evaluated the relative expansion of individual unit-cell parameters upon heating from 100 K to ambient conditions and compared the calculated data with those of the experiment. Results of this analysis are depicted in detail in Figure S18. The lowest thermal expansion can be observed for FFA_2_ in the directions of *b* and *c* vectors, and for IBU_S_ in the direction of *b* vector. These directions match the spatial orientation of the strongest hydrogen bonds in that crystal, which naturally restrain the crystal from expansion in these directions. Such crystals are then forced to expand in the other directions, where the cohesion is dominated by weaker dispersion interactions.^6^

To get a more global picture, we evaluated the linear expansion parameters in the individual crystallographic directions and compared their minimum and maximum values among the three directions for each target material. Results depicted in Figure S19 indicate that the purely PBE model M7 captures the extent of anisotropy relatively close to the experiment for all target structures, although it faces issues when π–π stacking dominates in one of the crystallographic directions. On the other hand, DFTB overstates the anisotropy for structures exhibiting rather isotropic thermal expansion. Concerning the composite M4 model, its ability to capture the anisotropy remains burdened by using the underlying DFTB unit-cell geometries. However, M4 performs in this context better than the original M1 model.

Absolute entropy is a property that is more sensitive to the phonon source, as depicted in Figure S20. For sulfathiazole, which is marginally impacted by the variation of *E*_el_(*V*) shapes between PBE and DFTB, methods M1, M2, and M5, all sharing the DFTB phonons, yield entropies systematically higher (by up to 8% or 25 J K^–1^ mol^–1^) than the remaining methods. This grouping cannot be observed for ibuprofen and flufenamic acid due to the interplay of the phonon and *E*_el_(*V*) effects on the entropy. Despite the attempts at parametrizing the DFTB model to capture the intramolecular frequencies in a more realistic way,^56^ the methods relying on PBE phonons yield entropies closer to the pseudoexperimental data. Calculated entropy values at 300 K are listed in Table S15.

**Figure 6:**
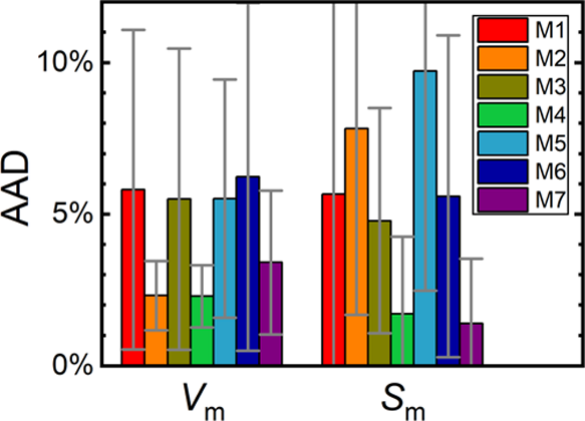
Absolute average deviations (AAD) of molar volumes (*V*_m_) and absolute entropies (*S*_m_) at 300 K and zero pressure calculated using individual formulations of the quasi-harmonic approximation (M1–M7, see Figure 2 for explanation) from the reference experimental data. Standard deviations of the errors of calculated properties that were observed for individual target crystal structures are depicted to illustrate the scatter of computational errors yielded by the considered methods.

A performance comparison of the individual combinations of DFTB and PBE methods that were used to calculate molar volumes and absolute entropies at 300 K and zero pressure is presented in Figure 6. For each target crystal structure with available reference data and each computational method, we evaluated the computational error from the experiment. For these sets of errors, we then evaluated their absolute average value (hereafter termed AAD) and their standard deviation (closely related to the scatter of errors). Those AAD values are averaged over data obtained for all seven structures for molar volumes, and for five structures for absolute entropies (excluding FFA_2_ and IBU_RS2_ due to reference data unavailability). Notably, raw PBE (=M7) yields appreciably lower AAD than raw DFTB (=M1) for both properties. Also, when compared to M1, the M7 method yields more consistent predictions with a lower scatter (by a factor of 2–3) of errors of *V*_m_ and *S*_m_ calculated for the individual target crystal structures.

Performance of the composite methods M2–M6 then varies strongly. To obtain an accurate prediction of *V*_m_ at 300 K, it proves to be sufficient to adjust the *E*_el_(*V*) minimum to the position given by M7. Methods sharing this location of the *E*_el_(*V*) minimum from PBE, namely, M2 and M4, yield very low AAD for *V*_m_ ranging within 2–3%. More laborious treatment of phonons does not bring any improvement over the initial M1 results when *E*_el_(*V*) remains to be treated with DFTB (as performed in M3 and M6). The composite method M4, being the logical option on how to efficiently improve both static and dynamic ingredients from the initial cheap DFTB method to an accurate but expensive PBE, then offers the lowest AAD for predicted *V*_m_.

Concerning the performance of *S*_m_ predictions, adapting solely the static or dynamic components of QHA does not bring any significant improvement over M1. It proves that only a concurrent adaptation of both terms within the M4 method leads to an important accuracy increase, lowering the AAD below 2%, being very close to the M7 results. Again, M4 and M7 methods offer the lowest scatter of errors observed for individual target crystal structures and thus the highest consistency of *S*_m_ predictions for various crystals.

We selected the experimental data as the primary reference level to benchmark our results against, as the former clearly correspond to the real behavior of materials and their properties. Keeping in mind that this comparison might lead to blurring some interference of errors related to individual components of the quasi-harmonic approximation, we also performed an additional comparison of the performance of all of the models with the highest-tier M7 one. Results of this additional analysis, depicted in Figure S21, indicate that the M4 model reproduces both the molar volumes and absolute entropies resulting from M7 most faithfully among the other given six models.

### 3.5 Response Properties

More stringent performance tests can be achieved by focusing on response properties that are related to second derivatives of the Gibbs energy with respect to temperature and/or pressure, such as the isobaric molar heat capacity (*C*_*p*,m_), coefficients of thermal expansivity (α_*p*_), or isothermic compressibility (*κ_T_*). Higher sensitivity of the second derivatives is the principal reason to expect higher deviation of the predictions from the experimental data.

**Figure 7:**
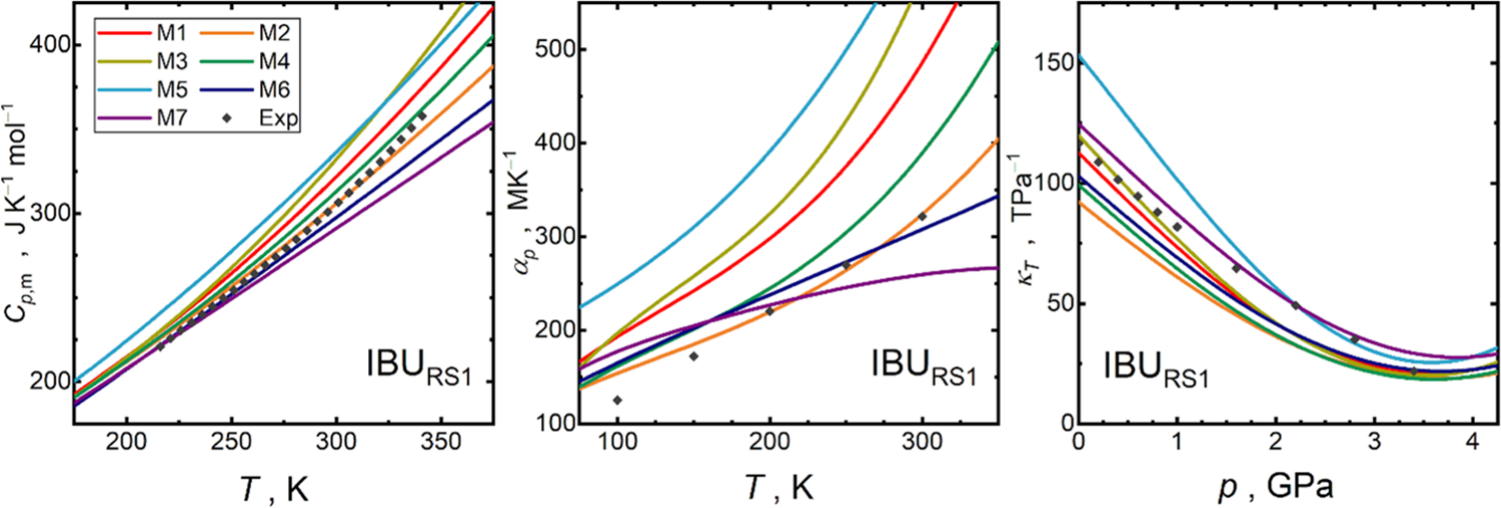
Isobaric molar heat capacity (*C*_*p*,m_) and coefficients of isobaric thermal expansivity (α_*p*_) and isothermal compressibility (*κ_T_*) for crystalline IBU_RS1_ calculated using individual formulations of the quasi-harmonic approximation M1–M7 (colored lines, see Figure 2 for explanation), and experimental reference data^38,39,71,72,75^ (gray diamonds).

Figures 7 and S22 depict a comparison of the calculated and experimental *C*_*p*,m_ data. Similar to the entropy results, heat capacity is primarily affected by the accuracy of the phonon frequencies. *C*_*p*,m_ curves for sulfathiazole (exhibiting similar *E*_el_(*V*) for all seven methods) are thus grouped at lower temperatures, with respect to the source of the underlying phonon data. All methods yield very similar *C*_*p*,m_(*T*) curves for flufenamic acid, systematically underestimating the actual *C*_*p*,m_ level but capturing the real slope well. A significant scatter of *C*_*p*,m_ for ibuprofen is observed among the methods, arising primarily from the impact of thermal expansion on the phonon properties. In the case of both ibuprofen and sulfathiazole, M1 leads to an overestimation of *C*_*p*,m_, whereas M7 systematically underestimates it.

This behavior further justifies the search for a viable composite approach that would provide *C*_*p*,m_ between the results of both raw methods, being even closer to the experimental data. Results of *C*_*p*,m_ from the M4 model are then close to capturing the experimental data most accurately out of all models in terms of both the magnitude and slope. Calculated *C*_*p*,m_ values at 300 K are listed in Table S16.

Relatively scarce experimental data do not allow for that extensive performance validation on the expansivity and compressibility coefficients, but still, important trends can be glimpsed. Figures 7 and S23 depict a comparison of calculated *α_p_* and *κ_T_* data with the experiment. Models M7 and M4 exhibit a reasonable agreement of *α_p_* over the following temperature range, although their slopes differ from the experiment. Other composite methods yield wildly scattered *α_p_* curves with no predictable systematic impact of substitution of either the static or the dynamic QHA components. Interestingly, the case of IBU_RS1_ shows that the overall accuracy of the predicted compressibilities is higher, with percentage deviations from experimental *κ_T_* at 300 K and 1 GPa amounting to −10, −21, and −6% for models M1, M4, and M7, respectively. To conclude, it is easier to capture from first principles a pure second derivative , being related to *κ_T_*, or  related to *C*_*p*,m_, rather than the mixed second derivative  that governs *α*_p_.

**Figure 8:**
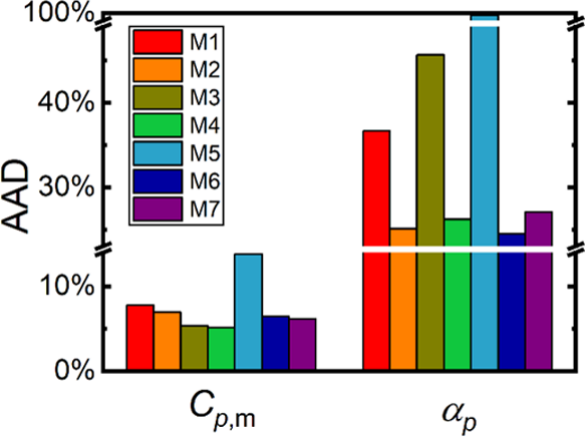
Absolute average deviations (AAD) of isobaric molar heat capacities (*C*_*p*,m_) and isobaric thermal expansion coefficients (*α_p_*) at 300 K and zero pressure calculated using individual formulations of the quasi-harmonic approximation (M1–M7, see Figure 2 for explanation) from the reference experimental data.

A global comparison of the performance of the individual methods, averaging the results over five crystal structures in the case of *C*_*p*,m_, and over four structures in the case of *α_p_* is given in Figure 8. Most *C*_*p*,m_ data sets exhibit an AAD ranging within an interval from 5 to 7%, which can be accepted as a fair accuracy of the given first-principles QHA models of *C*_*p*,m_.^8^ Notably, AAD of the M4 method is again at the bottom of this interval, outperforming the other composite methods. A direct combination of DFTB3 phonons and the PBE *E*_el_(*V*) curve, performed in the M5 method, leads to an excessive thermal expansion, which also negatively affects the accuracy of *C*_*p*,m_ with AAD doubled when compared with the remaining methods.

As mentioned, the isobaric thermal expansion coefficients are burdened with the highest computational uncertainty among the observed properties. The best models approach the experimental value with an AAD value around 25–30%, where both M7 and M4 belong to the best performing methods.

Figure S24 depicts a comparison of how closely individual models approach the raw M7. Using this point of view, M4 also belongs to the models reproducing the M7 heat capacities and thermal expansivities at the closest. Together with comparison of molar volumes and entropies, these data indicate that the performance comparison of individual models and trends of their result do not change upon changing the reference level from the experimental data to the raw M7 model, which supports the successful validation of the M4 model that is supposed to mimic the raw M7 closely.

### 3.6 Polymorph Ranking

Three pairs of polymorphs among the target materials were ranked in terms of their enthalpies and Gibbs energies at finite temperatures. Both raw M1 and M7 models, as well as all of their composites, were compared to verify the suitability of the promising M4 approach for the purpose of polymorph ranking. A detailed comparison of this ranking is depicted in Figure S25. Recall that the experimental ranking can be achieved with an uncertainty ranging from a few tenths of kJ mol^–1^ in an optimistic scenario, as stated in Table 2. Temperature adjustments then inflate these experimental uncertainties to manifold values due to the impact of uncertainties of experimental heat capacities.

**Figure 9:**
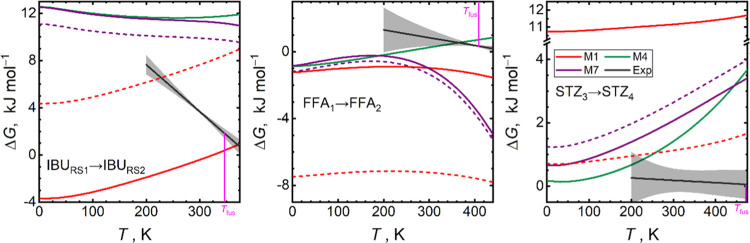
Gibbs energy change Δ*G* related to the phase transition between the target polymorphs of RS-ibuprofen, flufenamic acid, and sulfathiazole. Experimental data and their expanded uncertainties are depicted with gray lines and shaded areas, respectively. Single-point MP2C-F12/aug-cc-pVDZ refinement of proximate pair interaction energies in the crystal and MP2/aug-cc-pVDZ refinement of the monomer energies were applied in all cases. Results of raw PBE-D3/PAW and DFTB3-D4/3ob electronic energies without this refinement are given with dashed lines. Experimental fusion temperatures (*T*_fus_) of the highest-melting polymorph are given for reader’s convenience.

Figure 9 shows that both M1 and M7 methods predict the relationship of IBU_RS1_ and IBU_RS2_ polymorphs as monotropic with IBU_RS2_ being the metastable phase (Δ*G* is positive). This qualitatively matches the experimental observations at ambient pressure.^40^ DFTB geometries of the IBU polymorphs, adopted in methods M1, M3, and M6, surprisingly yield Δ*G* closer to the experimental value (ranging 3–4 kJ mol^–1^ at ambient conditions) than models relying on PBE geometries (depicted in Figure S25). The latter overestimate Δ*G* by a factor of 2–3. However, considering the steep slope of the experimental curve, its hypothetic extrapolation is likely to yield a Δ*G* very close to the PBE result at 0 K.

Slopes of the Δ*G*(*T*) curves are dominated by the entropy difference between polymorphs at individual temperatures, whereas their curvature is governed by the hierarchy of heat capacities between the two polymorphs. In this context, a steeper experimental Δ*G*(*T*) curve observed for racemic ibuprofen corresponds to an underestimation of the entropy difference between its polymorphs in the computational model.

A fragment-based refinement of the pair interactions within the crystal lattice at the MP2C-F12/aug-cc-pVDZ (for dimers) and MP2-F12/aug-cc-pVDZ (for monomers) levels of theory leads to a further 1.5 kJ mol^–1^ destabilization of IBU_RS2_ over the unrefined PBE results. Two aspects contribute to such a minor magnitude of the ab initio correction. First, the PBE and MP2 models predict only small differences of conformational energies of the monomers present in both polymorphs. Next, although the MP2C dimer corrections to the lattice energy of individual polymorphs are significant, such corrections largely cancel out within the ranked polymorph pair due to the similarity of the proximate molecular interactions in both polymorphs. Individual pair interaction energies are depicted in Figure S26, and thence resulting ab initio corrections to the lattice energies of individual polymorphs are listed in Table S17. Interestingly, the MP2C-F12/aug-cc-pVDZ refinement performed for DFTB geometries shifts the Δ*G* by massive 8 kJ mol^–1^ in the opposite direction. This highlights the importance of reaching high-quality unit-cell and conformational geometries for the polymorph ranking. It can then happen that too outlying energy terms yielded by DFTB would not be efficiently corrected by the fragment-based ab initio protocol.

DFTB phonon treatment states IBU_RS1_ as the higher-entropy polymorph, which translates to a trend of the respective Δ*G*(*T*) that is contradictory to the experiment for methods M1, M2, and M5, all relying on raw DFTB phonons. Adapting the DFTB phonons to approach the PBE model (in M3 and M4) already yields a correct entropy ranking of IBU_RS1_ and IBU_RS2_. The M4 model then qualitatively captures the Δ*G*(*T*) trend of the costly M7 model, at least below 250 K, which correctly retains the IBU_RS1_ as the lower-entropy polymorph at all temperatures below the melting temperature. These observations further justify the suitability of the composite M4 method for a more efficient polymorph ranking.

On the other hand, considering the high degree of similarity of both ibuprofen polymorphs, it is unlikely that both would differ in entropy as much (by about 40 J K^–1^ mol^–1^) to yield such a steep experimental Δ*G*(*T*) trend (being significantly steeper than the PBE results). This discrepancy raises the need for a future verification of the crystal structure of IBU_RS2_, its enthalpy of melting, or its potential disorder.

Figure 9 also shows that the current experimental data leave only a minimal chance for an enantiotropic relationship of FFA_1_ and FFA_2_ structures, favoring the thermodynamic stability of the FFA_1_ polymorph at ambient conditions in terms of its Gibbs energy. Both the experiment and PBE calculations present very small magnitudes of Δ*G* (below 1 kJ mol^–1^ in the absolute value at ambient conditions), indicating a large similarity of both polymorphs. Current M7 ranking offers a relatively satisfactory accuracy (calculated Δ*G* differs by less than 2 kJ mol^–1^ from the experiment under ambient conditions) in the context of the overall DFT performance. That is, however, insufficient to correctly assess relative stability of such similar polymorphs. The MP2C-F12 refinement of the M7 lattice energy shifts the Δ*G* term by about 0.4 kJ mol^–1^ in the right direction, however, being still not sufficient to yield the correct sign of Δ*G* at 0 K. More details on the underlying pair interactions and monomer energies in both flufenamic acid polymorphs are given in Figure S27 and Table S17.

At ambient conditions, PBE yields a qualitatively correct slope of the Δ*G*(*T*) trend, corresponding to FFA_2_ as the higher-entropy conformer at ambient to elevated temperatures. Interestingly, Δ*G*(*T*) obtained from the composite M4 approach follows the trend of its M7 analogue only at low temperatures below 200 K, not capturing the higher-entropy character of the FFA_2_ structure at elevated temperatures. Although the Δ*G*(*T*) profile resulting from the M4 model ranges at the closest to the experimental data, significance of this agreement should not be overestimated, as it is rather coincidental.

Finally, polymorph rankings of STZ_3_ and STZ_4_ present the hardest challenge as these polymorphs differ in their Gibbs energies by no more than 0.3 kJ mol^–1^. Our current experiments favor STZ_3_ as the more stable structure under ambient conditions with a rather monotropic relationship of the two. However, prevailing experimental uncertainties do not rule out the possibility for enantiotropy in this case. All M1–M7 methods capture the correct sign of the related Δ*G* and M7 results differ by no more than 3 kJ mol^–1^ from the experimental Δ*G*(*T*) curve. MP2C energy refinement for PBE geometries shifts the Δ*G* by 0.6 kJ mol^–1^ in favor of STZ_4_, with the dimer analysis presented in Figure S28 and the monomer energies listed in Table S17. It reveals that the currently used DFTB3-D4/3ob parametrization is not capable of reproducing the interaction energies of the closest sulfathiazole dimers in the crystal lattice, serving as an explanation for the anomalous behavior of the DFTB *E*_el_(*V*) curves. The composite M4 method again follows the M7 curve closely. All computational methods predict STZ_3_ to be the higher-entropy structure, translating to positive slopes of the computed Δ*G*(*T*) curves, which contradicts the experiments, suggesting a small entropy difference between both sulfathiazole polymorphs.

For all target polymorph pairs, only the M4 composite model yields consistent Δ*G* predictions. Most of the remaining considered composite approaches yield Δ*G*(*T*) results severely scattered in both magnitude and slope, without any systematic trends.

To provide insight on the sensitivity of the predicted Δ*G*(*T*) curves, we performed an additional analysis consisting in artificial manipulation of the original quasi-harmonic PBE-D3/PAW thermodynamic properties of STZ_4_ (but retaining STZ_3_ properties unchanged) and subsequent observation of the corresponding response of the Δ*G*(*T*) curves. Sulfathiazole represents the only case where all computational models invert the actual entropy hierarchy within the polymorph pair, propagating to an incorrect slope of calculated Δ*G*(*T*) curves. Therefore, we selected this system for such an analysis to demonstrate how subtle data alterations can modify the resulting trends on polymorph ranking.

First, we augmented the absolute entropy of the STZ_4_ phase by a constant value over the whole temperature interval. This corresponds to a hypothetic occurrence of a nonzero residual entropy of STZ_4_. We observed that adding roughly 8 J K^–1^ mol^–1^ to the entropy of STZ_4_ brings the slope of calculated Δ*G*(*T*) very close to that obtained from the experiment, as depicted in Figure S29. Note that this additional entropy value corresponds to a mere 3% of the actual entropy of sulfathiazole at ambient conditions, and it is lower than the configurational entropy limit for a three-state system, *R* ln(3).

Alternatively, we multiplied the heat capacity of STZ_4_ by a constant scale factor. This corresponds to the variation of the heat capacity within its computational uncertainty. In this case, we observed that augmenting the heat capacity of STZ_4_ by only 3% brings the slope of the Δ*G*(*T*) curve at ambient conditions very close to the experimental one, being depicted in Figure S30. Note that the uncertainty of heat capacities predicted by the M7 model ranges to around 7% for the given materials.

To conclude this sensitivity analysis, relatively modest alterations of the predicted thermodynamic properties of the ranked polymorphs can qualitatively change the shape of any Δ*G*(*T*) curve.

### 3.7 Accuracy-to-Cost Ratio

**Figure 10:**
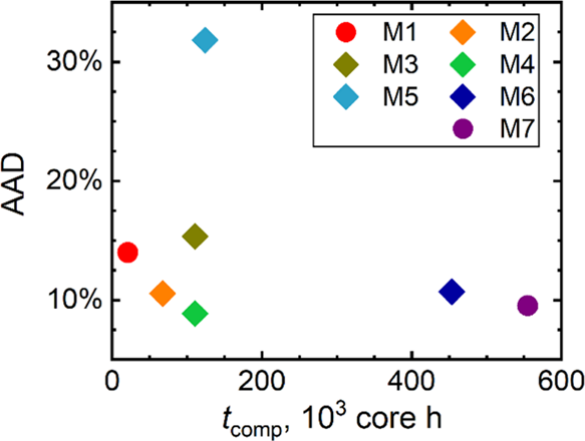
Illustration of the accuracy-to-computational cost ratio related to the individual raw and composite methods, combining the DFTB and DFT components of the quasi-harmonic processing.

To give a complete picture of the hierarchy of the initial raw M1 and M7 methods and their composite hybrids M2–M6, Figure 10 depicts the computational costs collected during all calculations for the IBU_RS1_ polymorph versus the mean of AAD data observed for *V*_m_, *S*_m_, *C*_p,m_, and α_p_ for all structures with available data. It confirms the expected superiority of the raw PBE (=M7) over the raw DFTB (=M1) in terms of accuracy, and it shows the order-of-magnitude higher computational complexity of M7. Most importantly, this accuracy-to-cost analysis confirms the leading role of the composite M4 approach, justifying its logical construction and verifying its maximal accuracy-to-cost ratio within the QHA framework. Note that the gap in the computational cost of PBE and DFTB calculations presented in Figure 10 will grow for systems larger than the IBU_RS1_ polymorph, which would further emphasize the benefits of using the composite M4 method, offering the M7 accuracy at a fraction of the M7 cost. Unlike the M4 model, direct combinations of DFTB and PBE ingredients of QHA, performed in M5 and M6 without any *E*_el_(*V*) shifts or phonon scaling, do not result in concurrently accurate and efficient models.

## 4 Conclusions

We demonstrated the limits of performing DFT-D predictions of cohesive properties at finite temperatures and finite pressures for large molecules with pharmaceutical significance in terms of the computational cost. To circumvent this issue and to extend the applicability range of quasi-harmonic first-principles modeling, we employed semiempiric quantum-chemical DFTB methods. These, however, cannot be fully relied on due to their lower accuracy. Therefore, we designed a composite approach, correcting both the static and dynamic components of the quasi-harmonic protocol so that the cheaply obtained DFTB results can be corrected from first principles at a modest additional cost to approach the accuracy of fully DFT-D methods. We demonstrated that this composite approach mimics the full PBE model at the closest, and it reproduces the experimental data with a very good accuracy-to-cost ratio. Although our benchmarking has been performed only for three archetypal active pharmaceutical ingredients and a particular pair of PBE-D3/PAW and DFTB3-D4/3ob levels of theory, applicability of the best performing composite approach is expected to be more general as it is also the logical option of how to combine the cheap DFTB and accurate DFT methods and their intermediate results. Such an achievement represents a massive step toward accurate high-throughput methods in the in silico polymorph ranking and toward computational screening of structure–property relationships for molecular materials built of large unit cells or bulky molecules, similar in size to those relevant for real pharmaceutical and optoelectronic applications. Implementation of a mode matching protocol assessing the covariance of eigenvectors of individual phonon modes within the extrapolative correction and quantification of its impact on the numerical accuracy leave space for future research in this direction.

## Supporting Information Available

Details on the computational and experimental setup; discussion on experimental reference data; additional results of the quasi-harmonic models; and ab initio analysis of pair interaction energies (PDF)
