# Supplementary material for: First-Principles Models of Polymorphism of Pharmaceuticals: Maximizing the Accuracy-to-Cost Ratio
Source: J Chem Theory Comput. 2024 Mar 26;20(7):2858–70. doi: 10.1021/acs.jctc.4c00099 (PMC11008097; doi:10.1021/acs.jctc.4c00099)
Supplement: Supplementary file 1 — ct4c00099_si_001.pdf [file ct4c00099_si_001.pdf]

First-principles Models of Polymorphism of Pharmaceuticals: Maximizing the Accuracy to Cost Ratio

Supporting Information

Jan Ludík[†], Veronika Kostková[†], Štefan Kocian[†], Petr Touš[†], Vojtěch Štejfa[†], Ctirad Červinka[†]*

[†] Department of Physical Chemistry, University of Chemistry and Technology Prague, Technická 5, CZ-166 28 Prague 6, Czech Republic

*Corresponding author: cervinkc@vscht.cz

1. Computational setup

This section provides details on how individual components of the quasi-harmonic processing were combined to model finite-temperature and finite-pressure properties of the seven target crystal structures, which are depicted in Figure S1 and described in Table S1 and Table S2. Table S3 provides details on the computational setup of the raw M1 and M7 quasi-harmonic approaches, whereas the remaining composite approaches M2 to M6 are described in Table S4.

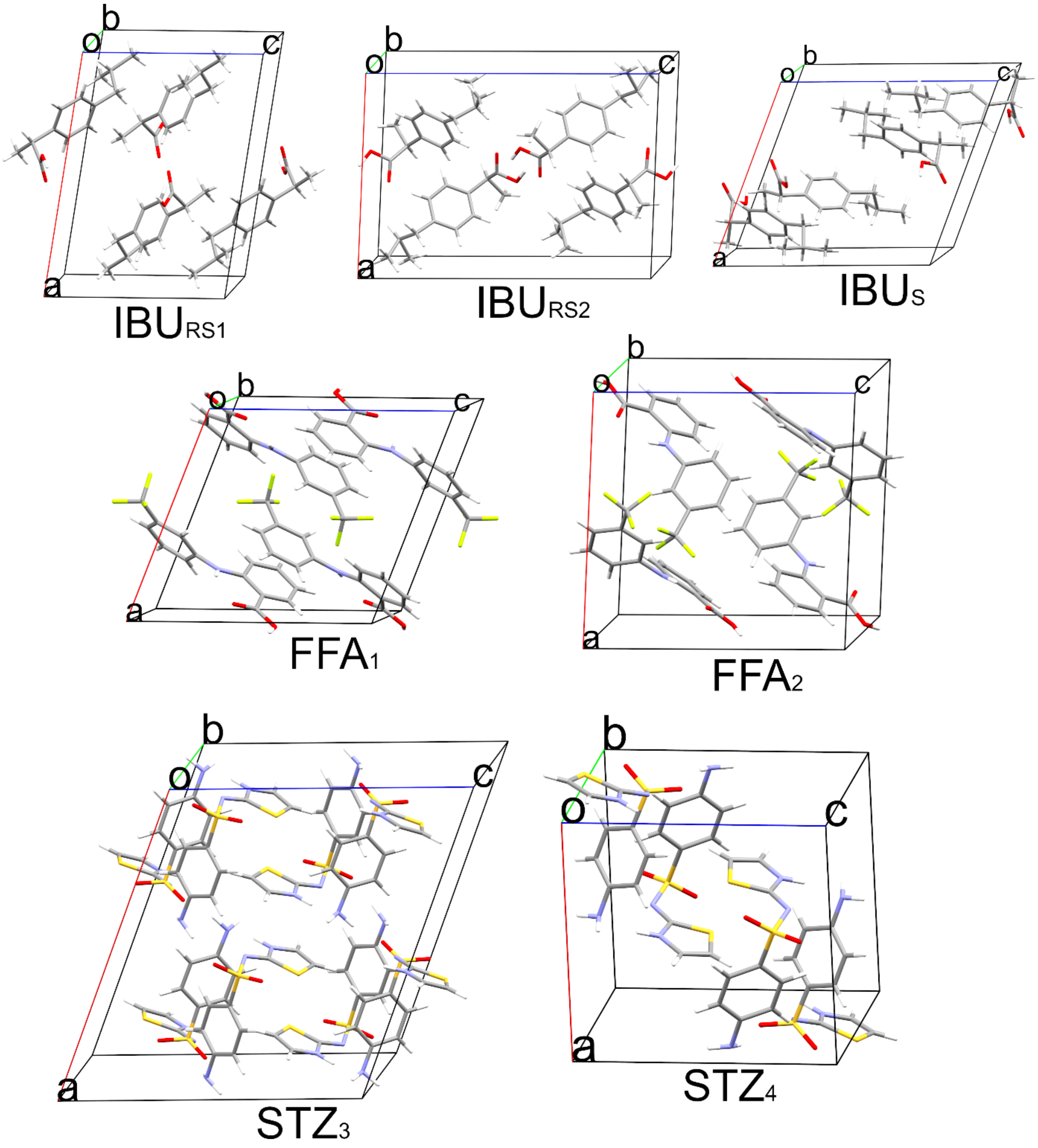

Figure S1. Illustration of target crystal structures of model APIs used for validation of computational methods throughout this work.

Table S1

Experimental unit-cell parameters (given in Å and deg.) of the target crystal structures determined in the literature at reference temperature T_{ref} (in K).

Crystal	CSD Refcode	T_{ref}	*a*	*b*	*c*	*β*	*Z*	Space group
IBU_{RS1}	IBPRAC16[1]	100	14.485	7.832	10.491	99.74	4	$P2_1/c$
IBU_{RS2}	IBPRAC04[2]	258	12.379	5.872	17.562	94.87	4	$P2_1/c$
IBU_S	JEKNOC12[3]	99	12.109	7.960	13.362	111.95	4	$P2_1$
FFA_1	FPAMCA17[4]	95	10.881	10.237	11.749	111.32	4	$P2_1/c$
FFA_2	FPAMCA18[5]	90	12.416	7.753	12.647	94.69	4	$P2_1/c$
STZ_3	SUTHAZ35[6]	100	17.417	8.491	15.495	112.76	8	$P2_1/c$
STZ_4	SUTHAZ45[6]	100	10.769	8.461	11.374	91.63	4	$P2_1/n$

Table S2

Dominant types of non-covalent interactions along the three crystal lattice vectors in the target crystal structures.

Crystal	Direction *a*	Direction *b*	Direction *c*	Anisotropy
IBU_{RS1}	H-bonding (COOH) + dispersion	H-bonding (COOH) + dispersion	H-bonding (COOH) + dispersion	Weak
IBU_{RS2}	H-bonding (COOH) + dispersion	H-bonding (COOH) + dispersion	H-bonding (COOH) + dispersion	Moderate
IBU_S	H-bonding (COOH)	H-bonding (COOH)	dispersion	Moderate
FFA_1	π–π stacking	H-bonding (COOH)	CF_3…CH dispersion	Moderate
FFA_2	π–π stacking	H-bonding (COOH)	H-bonding + dispersion	Strong
STZ_3	H-bonding (NH)	H-bonding (NH_2)	H-bonding + dispersion	Weak
STZ_4	SO_2…heterocycle dispersion	H-bonding (NH_2)	H-bonding (NH)	Moderate

Table S3

Detailed description of how the DFT and DFTB levels of theory were realized in the available computational software, including the important convergence settings.

Electron structure theory	**PBE-D3(BJ)/PAW**	**DFTB3-D4/3ob**
Software	VASP, version 5.4.4	DFTB+, version 21.1
Discrete points for $E_{el}(V)$ construction	15	30
Convergence criterion for basic geometry optimization	10^{-6} eV	10^{-5} Hartrees per·Bohr
Volume points where phonons were modeled	5	6 to 8
Convergence criterion for pre-phonon geometry optimization	10^{-8} eV	10^{-5} Hartrees per·Bohr

[a] For DFTB calculations, a set of D4 dispersion parameters recommended for the 3ob parametrization (a1, a2, s6, s8, s9) = (0.5523240, 4.3537076, 1, 0.6635015, 1) was used.[7]

Table S4

Definition of the composite quasi-harmonic methods developed in this work along with the color-coding occurring throughout the methods validation in the main paper. Individual methods combine results obtained with DFTB3-D4/3ob (abbreviated here as DFTB) and PBE-D3(BJ)/PAW (abbreviated as PBE).

	Static contributions: electronic energy			Dynamic contributions: phonon characteristics		
Method label	Raw $E_{el}(V)$ source[a]	Target E_{el} minimum[b]	$E_{el}(V)$ shifted[c]	Phonon trend source[d]	Target phonon reference[e]	Phonons extrapolated[f]
**M1**	**DFTB**	**DFTB**	**No**	**DFTB**	**DFTB**	**No**
**M2**	**DFTB**	**PBE**	**Yes**	**DFTB**	**DFTB**	**No**
**M3**	**DFTB**	**DFTB**	**No**	**DFTB**	**PBE**	**Yes**
**M4**	**DFTB**	**PBE**	**Yes**	**DFTB**	**PBE**	**Yes**
**M5**	**PBE**	**PBE**	**No**	**DFTB**	**DFTB**	**No**
**M6**	**DFTB**	**DFTB**	**No**	**PBE**	**PBE**	**No**
**M7**	**PBE**	**PBE**	**No**	**PBE**	**PBE**	**No**

[a] Initial construction of the entire $E_{el}(V)$ curve.
[b] Calculation of a single optimized unit-cell volume only, corresponding to the minimum of a $E_{el}(V)$ curve provided by the target method.
[c] Whole raw $E_{el}(V)$ curve shifted horizontally to place its minimum to the minimum coordinate observed for the target method.
[d] Initial calculations of the phonons vs. volume trends.
[e] Calculation of phonon characteristics at a single unit-cell volume only, typically corresponding to the minimum of the target $E_{el}(V)$ curve.
[f] Phonons vs. volume trends constructed using the target phonon reference at a single volume and Grüneisen parameters determined from the phonon source method.

2. Experimental setup

2.1. Materials

Description of samples treated in this work is given in Table S5. They were used without any purification and their purity was verified by the van't Hoff method.

Table S5

Sample descriptions.

Chemical name	CAS RN	M / g mol^{-1}	Brand	Purity according to producer	Mole fraction purity
Flufenamic Acid	766-49-4	281.23	Supelco	99 % [a]	0.998 [b]
Sulfathiazole	72-14-0	255.32	Supelco	100.3 % [c]	0.995 [b]

[a] Thin-layer chromatography.
[b] Determined from van't Hoff plot of the DSC measurements. Sulfathiazole decomposes upon melting, purity evaluated by the DSC should be considered a lower bound of the true value.
[c] Assay by electrophoresis.

2.2. X-ray powder diffraction

Identity of the crystal forms was verified by X-ray powder diffraction (XRPD) and the related diffractograms were analyzed with the software HighScore Plus in combination with annually updated powder diffraction databases PDF4+ and PDF4/Organics. The XRPD analysis was performed using a θ-θ powder diffractometer X'Pert^3 Powder from PANalytical in Bragg-Brentano para-focusing geometry using wavelength CuKα radiation (λ = 1.5418 Å, U = 40 kV, I = 30 mA). The samples were scanned at temperature (298.15 ± 3) K in the range of 5° to 50°2θ with a step size of 0.039°2θ and 0.7 s for each step.

2.3. Phase behavior study

The phase behavior was investigated with a heat-flux differential scanning calorimeter (TA DSC2500, TA Instruments, USA) whose temperature and enthalpy calibration was performed using peak areas of melting endotherms and onset melting temperatures of 1,3-difluorobenzene (Sigma-Aldrich, molar purity 0.9995), n-octane (Supelco, molar purity 0.997), indium, tin (both from a set of calibration substances for temperature and heat calibration of DSC, H. K. Cammenga, GEFTA, molar purities >0.9999), distilled and demineralized water (Millipore RQ, France, molar purity 0.9999), naphthalene (Aldrich 99+%, purified by zone refining, molar purity 0.9999), and benzoic acid (NIST SRM 39j, vacuum-dried, molar purity 0.9999) for several respective heating rates. Note that 1,3-difluorobenzene should be used as a DSC calibration standard with caution as it has two polymorphs with close melting points.[8]

If not stated otherwise, the results presented were obtained at a heating rate of 5 K min^{-1}. Sample loads of 5 to 10 mg were determined by an analytical balance with a readability of 0.01 mg, which was periodically calibrated. The overall molar purity of the samples was determined from the DSC thermograms using the van't Hoff method. Onset slope used for correction for the nonzero heating rate was determined as an average from calibration measurements for water, naphthalene, indium, and tin. The estimate of the standard uncertainty of molar purity x is $u(x) = 0.15(1-x)$.

2.4. Heat capacity measurements

The heat capacity measurements were carried out in the temperature range from 240 to 350 K using a Tian-Calvet calorimeter Microcalvet (SETARAM, France) employing the continuous heating method[9] with a three-step methodology.[9] The following steps were performed to obtain the heat capacity of a sample: i) measurement of the sample, ii) measurement of the reference material (synthetic sapphire, NIST standard reference material No. 720), and iii) measurement with both cells empty (so-called blank experiment). A detailed description of the performance of the Microcalvet calorimeter and calibration results are provided in ref. [10]. Based on the calibration measurements, the combined expanded uncertainty $U_c(C^{o}_{p,m})$ (at the 0.95 level of confidence) of the heat capacity measurements with the given methodology was estimated to be $U_c(C^{o}_{p,m}) = 0.006\ C^{o}_{p,m}$.

3. Thermodynamic data obtained in this study

In the following text, an alternative notation of individual polymorphs is used. As explained in Table S6, notation crI is equivalent to FFA_1 in discussion relevant for flufenamic acid, and crIII is equivalent to STZ_3 in the context of sulfathiazole.

3.1. Phase behavior of flufenamic acid

Flufenamic acid has a complex phase behavior, which has been extensively studied and is relatively well described,[4] except for few issues. Perhaps the most critical is the inconsistency between Table 6.1 and Figure 6.1 in the SI of ref. [4], which are in conflict with respect to order of melting temperatures of forms II and III. Based on comparison with our results and previous literature,[11-12] it seems that form III melts lower than I and II. At the same time, it has a higher melting enthalpy than form I, which means that the forms are enantiotropic and at low temperatures, form III should become thermodynamically stable, as confirmed in ref. [12].

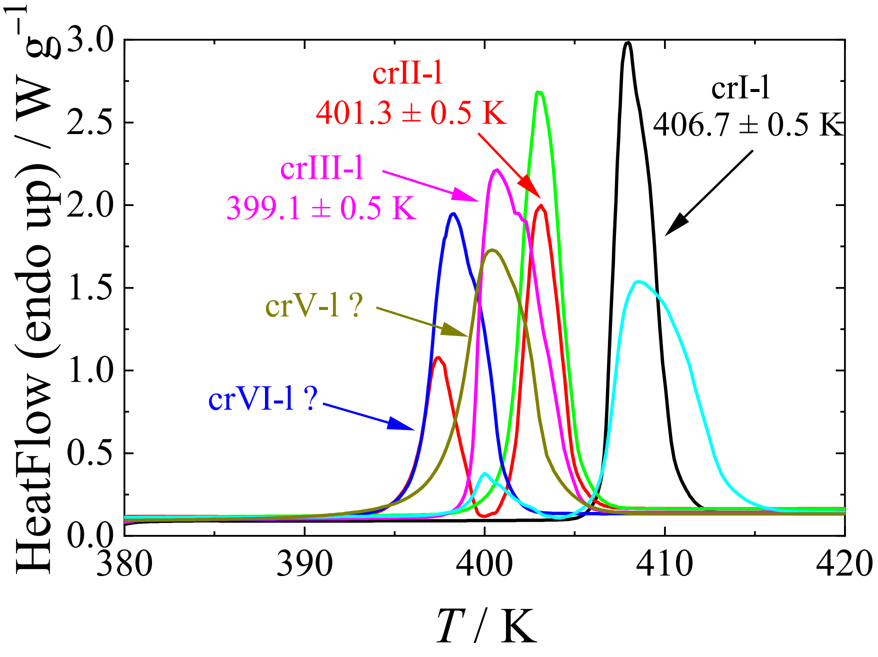

Figure S2. Typical thermograms observed for flufenamic acid.
Starting form: —, "as received" material – form I, —, form II, —, form III (or its mixture with form II), —, form V, —, form IV, —, mixture III+I, —, mixture VI+II. All experiments are recorded at a heating rate of 5 K min^{-1}, except for form V (olive line) and mixture III+I (cyan line), which are at 10 K min^{-1} and were scaled by a factor of 1/2 for clarity.

It was observed in this work that the flufenamic acid slowly decomposes at temperatures around its melting point (onset temperatures and enthalpies slightly decreased between consecutive

runs), which slightly lowers the achievable uncertainty of the DSC results. In case of fast cooling, the sample has not crystallized completely and a glass transition could be observed at 283.1 ± 0.4 K at a heating rate of 5 K min^{-1}. This value is in a reasonable agreement with the Beaman-Kauzmann rule yielding $T_g/T_m \approx 0.70$ for the high-melting polymorph crI.

Table S6

Phase behavior of selected polymorphs of flufenamic acid and sulfathiazole

Phase	Preparation	Transition to	This work [a] T / K	This work [a] ΔH^0_m / kJ mol^{-1}	Literature T / K	Literature ΔH^0_m / kJ mol^{-1}
			Flufenamic acid			
FFA_1 = crI	Received material	l	406.7 ± 0.5	27.8 ± 0.9	406 (ref. [11])	
					407.7 (ref. [4])	27.6 (ref. [4])
FFA_2 = crII[b]	Slow cooling of melt to 333 K in the DSC	l	401.3 ± 0.5	25.1 ± 1.5	401 (ref. [11])	
					402.8 (ref. [4])	24.7 (ref. [4])
FFA_3 = crIII[c]	Fast cooling of the melt to 298 K	l	399.1 ± 0.5	28.2 ± 0.9	398 (ref. [11])	
					400.6 (ref. [4])	29.7 (ref. [4])
		crI	~356	1.0 ± 0.2	315.2 (ref. [12])	
			Sulfathiazole			
STZ_3 = crIII	Received material	crI	-	-	423-443 (ref. [13])	
					427 (ref. [14])	4.9 (ref. [15])
					415.5 (ref. [15])	
		crV[d]	~434	6.6 ± 0.3		
STZ_5 = crV	Annealed at 435 K	l	469.0 ± 0.3	28.8 ± 0.8	469.7 (ref. [15])	31.6 (ref. [15])
STZ_1 = crI	Annealed at 470 K	l	474.2 ± 0.3	28.0 ± 0.8	475.0 (ref. [15])	27.9 (ref. [15])
					474 (ref. [13])	
					475 (ref. [14])	
STZ_4 = crIV[e]	Recrystallized from water (ref. [15])	crV[d]	~428	6.2 ± 0.3	434 (ref. [15])	6.4 (ref. [15])

[a] DSC results are given with their expanded uncertainties (k = 2). Corrections to melting temperatures for the impurity content are included.

[b] Form prepared only in DSC experiments, identity assigned based on agreement of T_{fus} and ΔH^0_m with literature.

[c] Form III with about 13 % of phase I according to XRPD.

[d] Transformation proceeds in three steps, the reported values correspond to the largest peak. It is not clear, if the minor peaks correspond to consecutive transformations through other forms or to transformations of trace forms to crV or to crI.

[e] Form IV with about 15 % of phase III according to XRPD. Small amount of residual water (w_{H2O} = 0.0002) was detected by a eutectic peak close to 273 K.

The “as received” form of flufenamic acid was I. Form III was prepared by melting the material at 433 K and left to cool to room temperature spontaneously. According to XRPD, this sample contained 13% of form I. Upon heating in DSC, the same sample exhibited an endothermal solid-solid transition before melting as form I, which is in agreement with the enantiotropic

relationship of phases III and I. Other phases were not successfully prepared in larger amounts, only within the DSC experiments. Form II (identified based on its melting temperature and enthalpy) was observed to crystallize in some runs with slow cooling of the melt. Some other forms were observed during the experiments (probably V and VI), but they could not be unambiguously identified because of close melting temperatures and fusion enthalpies with form IV.

The typical thermograms observed for flufenamic acid including some runs with concomitant occurrence of more forms are visualized in Figure S2. Melting temperatures and fusion enthalpies of forms I, II and III are compared with literature in Table S6. Melting temperatures observed in this work are in good agreement with ref. 11, but they are about 1.5 K lower than in ref. 4. Since our calibration procedure comprises of seven compounds and the presented results are corrected for impurity content, it seems more probable that the systematic difference is introduced by the literature values.

3.2. Phase behavior of sulfathiazole

Sulfathiazole was previously shown to form five crystal structures and the ways for their preparation were described.[15] According to our observations, sulfathiazole abruptly decomposes in liquid form at temperatures above approximately 445 K. When the melt is cooled to lower temperatures, the decay stops, but the sample was never observed to crystallize. The observed glass transition temperatures varied between 329 and 346 K for various samples, probably depending on their purity. The lower results would be in agreement with the Beaman-Kauzmann rule with $T_g/T_m \approx 0.69$, but not the higher ones. We assume, that because of the instability of the melt, it not possible to determine the glass transition temperature of sulfathiazole accurately with the conventional technique.

The "as-received" form of sulfathiazole was crIII, which is supposedly thermodynamically stable at the room temperature. Unlike in the literature reports, form III transformed to crV upon heating at around 435 K. The large peak of quantitative transformation was accompanied with two minor peaks, which might either mean that the sample does not recrystallize directly from III to V, but through a sequence of several transformations, or that these correspond transformations of trace forms contained in the sample to crV or to crI. Upon additional cooling, form crV incongruently melts at 469.0 ± 0.3 K and gives form I, which melts at 474.2 ± 0.3 K. Preparation of a larger amount of form V was attempted by annealing the material at 455 K, but the XRPD analysis corresponded to a mixture of V and I. Because of the incongruent melting, it is not possible to verify or refute the concomitant occurrence of V and I in the DSC

experiments. On the other hand, the peak of III-V transformation has a regular shape, which suggests a simple recrystallization process. Because of their close fusion enthalpies, it is not definite if forms I and V are monotropic or enantiotropic, but at least at elevated temperatures, form V is less stable than I and a spontaneous recrystallization may proceed between the two forms. Larger amount of form I was prepared by annealing the material at 470 K and according to XRPD, the sample was crystallographically pure, although it turned slightly grey because of thermal decomposition.

Form IV was prepared according to the procedure suggested by Abu Bakar et al.[15], by cooling recrystalization from water. Crystal stucture of the prepared sample was verified by XRPD with a possible contamination by 15% of form III, as is common according to the literature.[13] DSC experiments with this sample showed very similar behaviour as form III did. The sample recrystallized to form V showing three peaks, of which the largest was located at lower temperature than in case of form III and was also slightly smaller. Forms III and IV are thus very close both from the crystallographic and thermodynamic point of view.

The typical observed thermograms are visualized in Figure S3. Phase-transition temperatures and enthalpies of the observed forms are compared with the literature in Table S6.

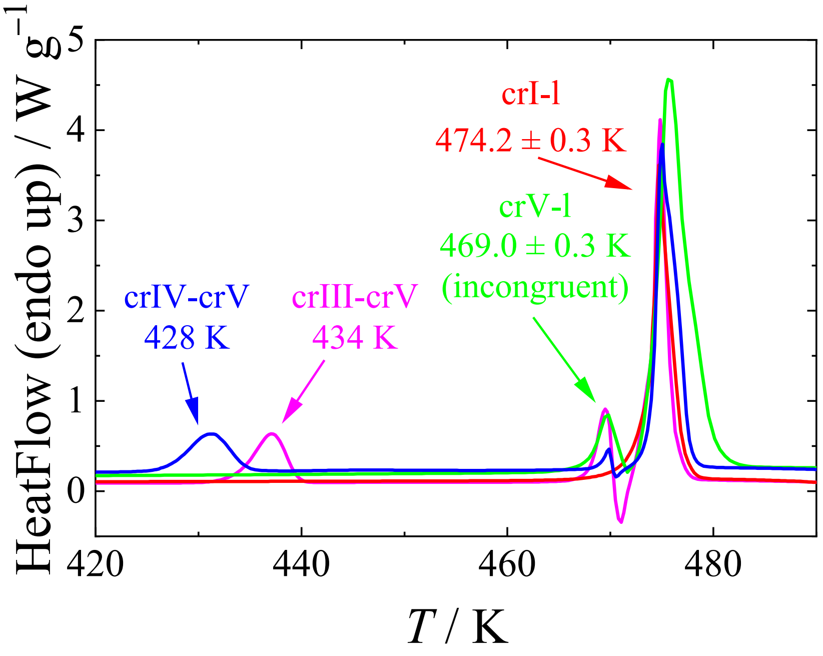

Figure S3. Typical thermograms observed for Sulfathiazole.

Starting form: —, “as received” material – form III, —, form I, —, form IV, —, form V. All experiments are recorded at a heating rate of 5 K min^{-1}, except for form V (green line), which is at 10 K min^{-1}.

3.3. Heat capacities

Heat capacities of forms of flufenamic acid and sulfathiazole that were successfully prepared in larger amounts are given in Table S7. According to the XRPD analysis, form III of flufenamic acid contained about 13% of form I and form IV of sulfathiazole about 15 % of form III. Since heat capacities of these concomitant forms are practically identical, no corrections were performed. In one experiment, heat capacities of mixture of forms V+I of flufenamic acid were determined. According to XRPD analysis performed six days later, the composition was 56% of form I and 44% of form V. The heat capacities of form V in Table S7 were calculated by linear extrapolation with the assumption that this composition was constant. However, the sample more probably continuously recrystallized from form V to more stable form I and the real heat capacity of form V can be in between the datasets labeled 'form I+V' and 'form V'.

The experimental heat capacities were represented with a polynomial equation,

$$C_{p,\mathrm{m}}^{\mathrm{o}} / \left(\mathrm{J\ K^{-1}\ mol^{-1}}\right) = a + b\left(\frac{T}{100\ \mathrm{K}}\right) + c\left(\frac{T}{100\ \mathrm{K}}\right)^2 \quad (1)$$

the parameters of which are given in Table S8. Heat capacities of form I and form III of flufenamic acid are compared in Figure S4. Form III has steeper temperature dependence of the heat capacity curve, which correlates with the assumption that the two phases are enantiotropic. Heat capacities of different forms of sulfathiazole are compared in Figure S5. Forms III and IV that have similar crystallographic patterns and close melting properties have also equal heat capacities within the uncertainty of the measurement. Form I of sulfathiazole that is enantiotropic with respect to forms III and IV and has a significantly higher fusion entropy also exhibits higher heat capacity over the whole range of temperatures. At high temperatures, the heat capacity curves approach each other and should intersect around 383 K (I and III) or 405 K (I and IV), which is still below the observed III-I and IV-I transition temperatures. This observation is again in agreement with the enantiotropic relationship between forms I and III and forms I and IV. Form V of sulfathiazole was only studied in a mixture with form I, and there is a lower level of certainty about its heat capacities. Its heat capacities seem to be lower than that of form I, but higher than that of form III and IV. As in the case of form I, they seem to intersect with heat capacities of forms III and IV at elevated temperatures.

Table S7

Experimental heat capacities of flufenamic acid and sulfathiazole (in J K^{-1} mol^{-1}) [a]

	Flufenamic acid		Sulfathiazole				
T / K	form I	form III [b]	form I	form III	form IV [c]	form I+V [d]	form V [e]
240	255.5			212.5			
245	260.1			216.2			
250	264.7			220.3			
255	269.3			224.7			
260	273.8	269.0	241.0	228.7	225.5	237.5	233.3
265	278.4	275.0	245.8	232.7	230.6	241.8	236.9
270	282.8	280.5	250.1	236.7	235.1	245.9	240.7
275	287.4	285.5	254.3	240.6	239.6	249.9	244.6
280	291.9	290.5	258.4	244.6	243.6	253.9	248.4
285	296.5	295.4	262.4	248.5	247.7	257.8	252.2
290	301.0	300.2	266.3	252.4	251.7	261.6	255.8
295	305.5	305.0	270.2	256.3	255.7	265.4	259.6
300	310.0	309.6	274.0	260.2	259.7	269.2	263.3
305	314.5	314.3	277.6	264.1	263.6	272.8	267.0
310	318.9	319.0	281.2	268.0	267.5	276.4	270.7
315	323.4	323.5	284.6	271.8	271.3	280.0	274.3
320	327.8	328.2	287.9	275.6	275.2	283.5	278.1
325	332.2	332.9	291.3	279.4	279.0	287.0	281.9
330	336.5	337.6	294.5	283.3	282.8	290.5	285.5
335	340.9	342.3	297.8	287.1	286.6	293.9	289.2
340	345.2	347.1	301.1	290.9	290.4	297.4	292.9
345	349.6	352.0	304.3	294.6	294.2	300.8	296.6
350	353.6	356.9	307.6	298.5	298.0	304.1	299.9

[a] The combined expanded (k = 2) uncertainty of the experimental heat capacity data is $U_c = 0.006\ C^{\circ}_{p,m}$.

[b] According to the XRPD, sample of form III contained up to 13% of form I. Since heat capacities of form III and I are practically identical, no corrections were performed.

[c] According to the XRPD, sample of form IV of sulfathiazole contained up to 15 % of form III. Since heat capacities of form III and IV are practically identical, no corrections were performed. Small amount of residual water (w_{H2O} = 0.0002) was detected through a eutectic peak close to 273 K.

[d] According to the XRPD performed six days later, the mixture contained 56% of form I and 44% of form V.

[e] Calculated from experimental heat capacities of form I and mixture (I+V) in previous columns.

Table S8

Parameters of equation 1 for isobaric heat capacity of the APIs

	Flufenamic acid		Sulfathiazole			
	form I	form III	form I	form III	form IV	form V
a	41.211	21.802	−93.358	25.397	−57.164	40.225
b	89.49	95.78	170.13	78.17	131.02	74.32
c	0	0	−15.910	0	−8.466	0

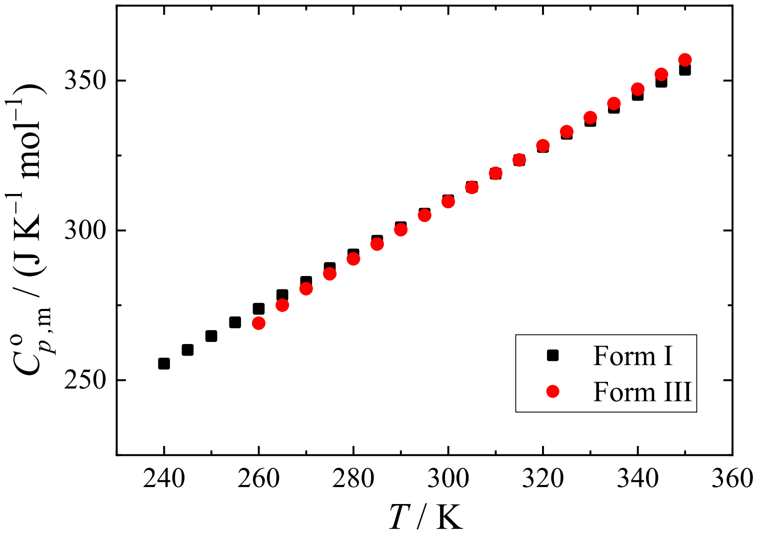

Figure S4. Heat capacities of flufenamic acid experimentally determined in this work.

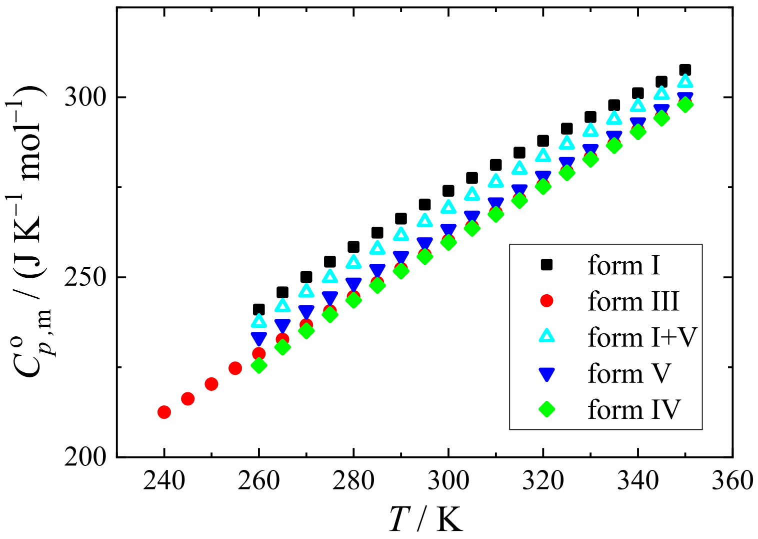

Figure S5. Heat capacities of sulfathiazole experimentally determined in this work.

Due to the from-below limited operating range of the Microcalvet calorimeter, we had to derive pseudo-experimental absolute entropies of the crystal phase from the raw experimental heat capacity and from the PBE-D3/PAW quasi-harmonic heat capacities. The experimental data were determined in this work (flufenamic acid and sulfathiazole) or taken from ref. 16 (ibuprofen). The calculation results were scaled by a constant factor below the lower limit of measurements (240 K for FFA_1 and STZ_3, 260 K for STZ_4, and 216 K for $IBU_{RS1}$and IBU_S) to match the experimental value at the minimal temperature, enabling a smooth joint of the two data sets as depicted in Figure S6. Absolute entropy profiles were then constructed via essential thermodynamic integration, assuming that there is zero residual entropy for these materials at 0 K. Given pseudo-experimental heat capacities are listed in Table S9 and entropies in Table S10.

Table S9

Pseudo-experimental[a] isobaric heat capacities of selected polymorphs of the target molecular crystals (in J K^{-1} mol^{-1}).

T / K	IBU_{RS1}	IBU_S	FFA_1	STZ_3	STZ_4
10	5.60	5.71	5.91	4.25	3.80
20	23.17	23.43	24.34	17.87	16.33
30	41.20	41.32	43.00	32.24	30.02
40	58.01	57.89	60.17	45.88	43.31
50	71.97	71.66	74.13	57.31	54.63
60	83.62	83.23	85.61	66.93	64.24
70	94.65	94.24	96.51	76.14	73.45
80	105.12	104.75	106.90	85.00	82.31
90	115.08	114.81	116.85	93.55	90.86
100	124.60	124.47	126.43	101.84	99.16
110	133.72	133.80	135.71	109.90	107.24
120	142.51	142.83	144.75	117.79	115.14
130	151.02	151.64	153.63	125.56	122.91
140	159.32	160.27	162.41	133.24	130.60
150	167.46	168.77	171.16	140.89	138.25
160	175.49	177.21	179.95	148.54	145.91
170	183.48	185.64	188.85	156.25	153.61
180	191.48	194.11	197.93	164.07	161.40
190	199.55	202.67	207.26	172.03	169.33
200	207.75	211.38	216.91	180.18	177.43
210	216.04	220.30	226.69	188.39	185.61
220	224.76	229.69	236.36	196.51	193.70
230	234.30	239.74	245.94	204.52	201.70

[a] Data are based on scaling the PBE-D3/PAW results (M7) by a factor ξ (given in Figure S6) to match the raw experimental data at the lower edge of the experimental range.

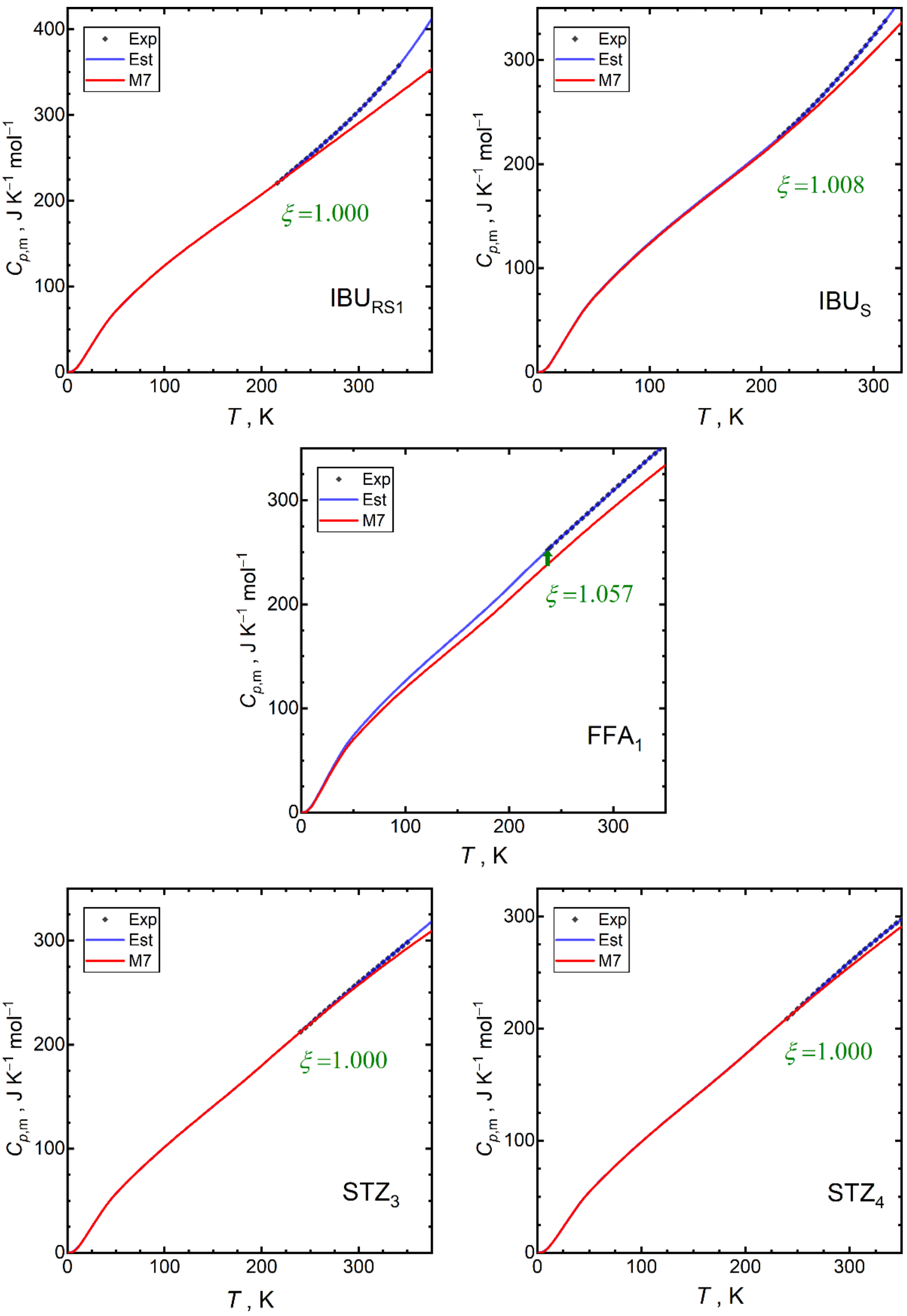

Figure S6. Illustration of the development of pseudo-experimental isobaric heat capacities (Est) based on scaling the PBE-D3/PAW results (M7) by a factor ξ to match the raw experimental data (Exp) at 240 K (the lower edge of the experimental range). Note that ξ is kept constant below 240 K but it becomes slightly temperature dependent above 240 K to account for the contributions to C_p from the thermal expansion.

Table S10

Pseudo-experimental[a] absolute entropies of selected polymorphs of the target molecular crystals (in J K^{-1} mol^{-1}) [a]

T / K	IBU_{RS1}	IBU_S	FFA_1	STZ_3	STZ_4
10	1.87	1.90	1.97	1.42	1.27
20	11.07	11.25	11.66	8.45	7.64
30	23.88	24.15	25.08	18.42	16.83
40	38.09	38.35	39.86	29.59	27.31
50	52.60	52.81	54.86	41.12	38.24
60	66.76	66.91	69.40	52.42	49.06
70	80.49	80.57	83.42	63.44	59.66
80	93.82	93.85	96.99	74.19	70.05
90	106.78	106.77	110.16	84.69	80.24
100	119.40	119.37	122.97	94.98	90.24
110	131.70	131.67	135.46	105.07	100.08
120	143.72	143.71	147.66	114.97	109.75
130	155.46	155.49	159.60	124.70	119.27
140	166.96	167.04	171.30	134.29	128.66
150	178.23	178.39	182.80	143.74	137.93
160	189.30	189.55	194.13	153.08	147.10
170	200.17	200.55	205.31	162.32	156.18
180	210.89	211.40	216.36	171.47	165.18
190	221.46	222.12	227.31	180.55	174.11
200	231.90	232.74	238.18	189.58	183.00
210	242.24	243.27	249.00	198.57	191.86
220	252.48	253.73	259.77	207.52	200.68
230	262.68	264.16	270.49	216.44	209.47
240	272.85	274.58	281.16	225.31	218.22
250	283.00	285.02	291.79	234.16	226.94
260	293.13	295.49	302.36	242.98	235.64
270	303.25	306.01	312.86	251.75	244.34
280	313.36	316.59	323.31	260.49	253.03
290	323.50	327.24	333.70	269.20	261.72
300	333.65	337.98	344.05	277.88	270.38
310	343.85	348.81	354.35	286.53	279.03
320	354.11	359.75	364.61	295.15	287.65
330	364.44	370.80	374.82	303.75	296.24
340	374.87	381.97	385.00	312.32	304.80
350	385.41	393.28	395.15	320.88	313.32

[a] Data are based on scaling the PBE-D3/PAW results (M7) by a factor ξ (given in Figure S6) to match the raw experimental data (at the lower edge of the experimental range).

3.4. Polymorph ranking

Experimental data on heat capacities and solid-solid and/or solid-liquid phase transition temperatures and enthalpies determined in this work were used to calculate profiles of the Gibbs energy change $\Delta_I^{\alpha}G_m^0(T)$, corresponding to transitions from the high-temperature polymorphs FFA_I and STZ_I to different phases. Figure S7 and Figure S8 also compare the current ranking with that arising from literature data on phase behavior of flufenamic acid and sulfathiazole, respectively.

Our results agree qualitatively well with the literature for flufenamic acid, both confirming that phases FFA_I and FFA_3 are the most stable at high-temperatures and ambient-temperatures. The most significant difference is the position of the triple point temperature of crI and crIII. According to the fusion enthalpies and melting temperatures observed in this work and in literature,[4] it should occur at around 260 K and 340 K, respectively. The difference is obviously given by the long temperature extrapolation, which exemplified the uncertainties in the experimental data. An enantiotropic phase transition of form III to form I at 315.2 K was described.[12] The triple point temperature must therefore be below 315 K, since the solid-solid transition can superheat noticeably (as e.g. in this work, where it was observed at 356 K, see Table S6), but may not occur below the equilibrium temperature. Mutual stability of forms III and I at 298 K thus remains unclear.

For sulfathiazole, very small differences among individual polymorphs make it difficult to establish a definite ranking of their stability. Whereas the phase STZ_I is clearly the high-temperature structure, current experimental uncertainties admit that either STZ_3 of STZ_4 is the most stable modification at ambient-temperatures. The most beneficial clue to prioritize one of the possibilities is the identity of the commercial form. Forms III and IV crystallize concomitantly in most cases,[15] but pure form III is commercially available, which renders very improbable that a pure metastable form, which is obtainable only with severe difficulties, would be distributed. On the other hand, slow recrystallization from the concomitant mixture of III and IV to the more stable form may easily occur during the storage and expedition. Stability ranking based on our experimental data (Figure S8a) is thus more probable.

To derive the polymorph differences in terms of enthalpy and Gibbs energy in Table 2 and in Figure S7 and Figure S8, only experimental phase transition temperature and enthalpy data were used. Operating range of the employed calorimeters does not enable to cover the whole interval from 300 K up to the melting temperatures. Furthermore, heat capacities of both

polymorphs within the ranked polymorph pairs are very similar (for STZ), or the higher-energy polymorph could not be prepared in a sufficient quantity to measure its heat capacity reliably (for FFA). However, we incorporated the expected uncertainties of the measured heat capacities to estimate the overall uncertainty associated to the temperature adjustment of ΔH and ΔG terms to 300 K.

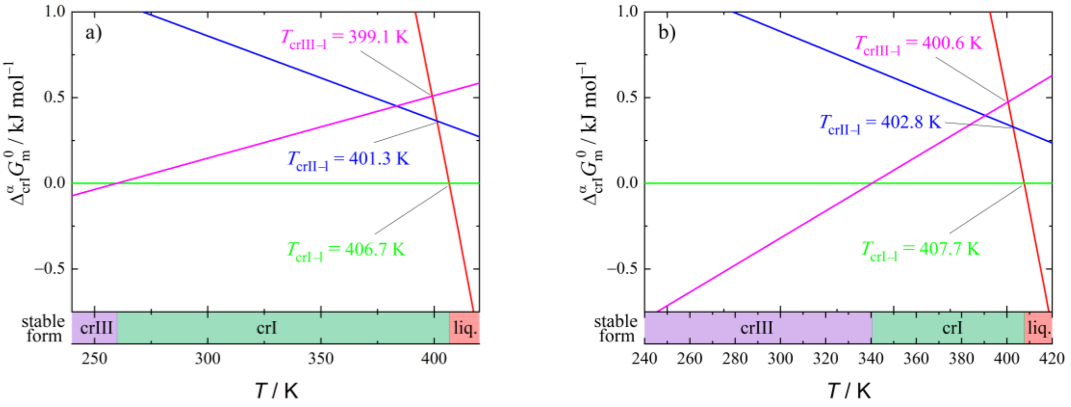

Figure S7. Experimental polymorph ranking for flufenamic acid: a) according to data observed in this work; b) according to phase transition parameters from the literature.[4] Legend: —, liquid, —, form I, —, form II, —, form III.

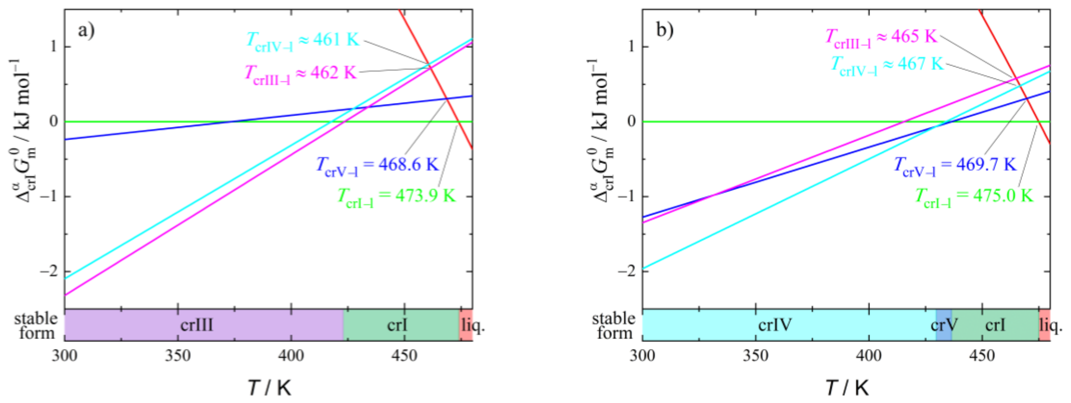

Figure S8. Experimental polymorph ranking for sulfathiazole: a) according to data observed in this work; b) according to phase transition parameters from the literature.[15] Legend: —, liquid, —, form I, —, form II, —, form III, —, form IV.

3.5. Densities of crystals

Density data were searched for all target materials in the Cambridge Structural Database. There are multiple entries for all the structures, except IBU_{RS2} polymorph. Figure S9 to Figure S11 depict that although there are crystal structures resolved at more than one temperature, the interlaboratory scatter of the unit-cell volume and related crystal densities can be significant. Therefore, we performed interpolations of the crystal densities with a cubic polynomial only for structures where data were available at least for three distinct temperatures, all consistently following a clear trend. That scenario was fulfilled only for IBU_{RS1}, STZ_3, and STZ_4, for which we were also able to derive experimental information on thermal expansivities. Data points outlying by more than 3 kg m^{-3} from the cubic interpolation were omitted. In a similar way, variable-pressure density data were interpolated for IBU_{RS1}. Note that for the sake of consistency of the thermal expansivity or isothermal compressibility data, it is safer to use multiple data points determined on a single apparatus over a wide temperature or pressure range, rather than data points at multiple conditions each obtained on a different apparatus. Pressure dependent data for IBU_{RS1} and their interpolation is given in Figure S12.

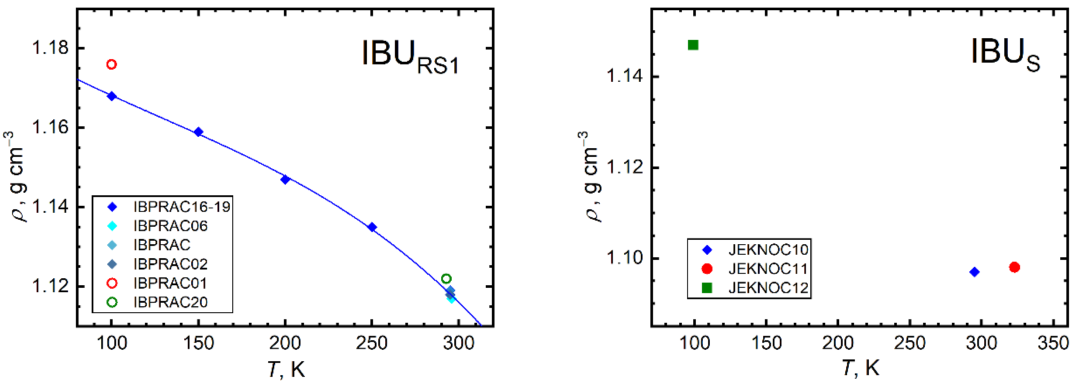

Figure S9. Experimental density data for two crystal forms of ibuprofen at ambient pressure, all culled from the respective CSD entries. A cubic fit of the variable-temperature density data showing consistency (full symbols) is depicted for IBU_{RS1}.

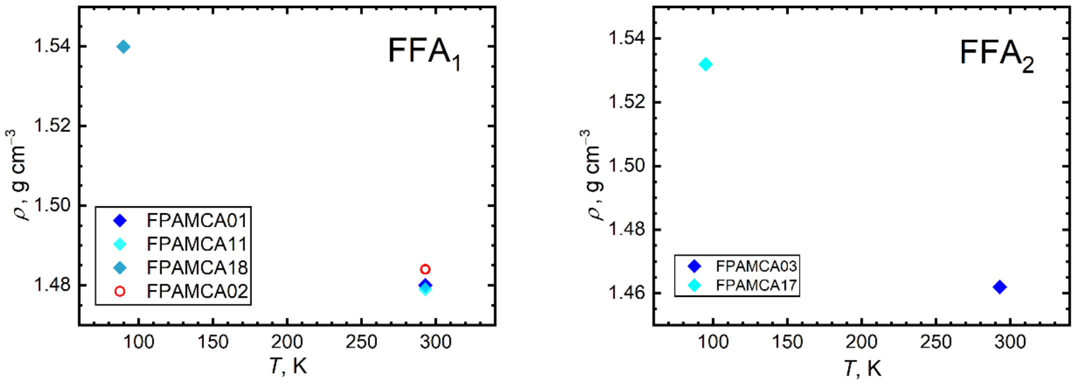

Figure S10. Experimental density data for two crystal forms of flufenamic acid, all culled from the respective CSD entries.

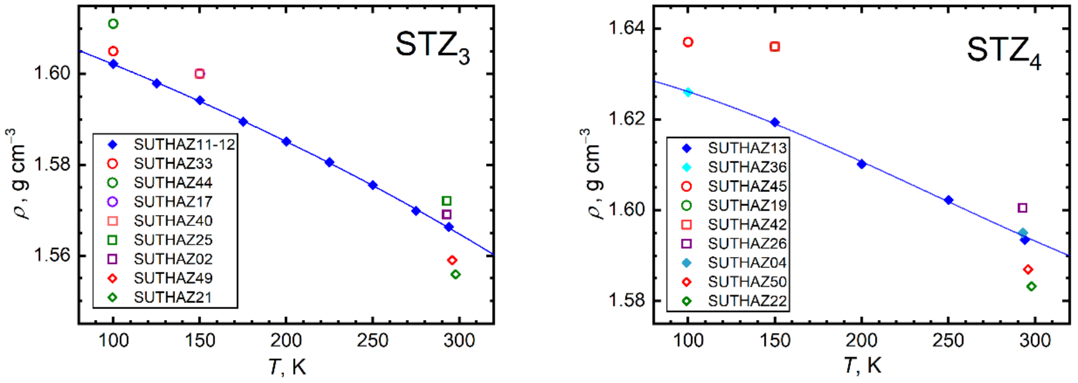

Figure S11. Experimental density data for two sulfathiazole crystal forms, all culled from the respective CSD entries. Cubic fits of the variable-temperature density data showing consistency (full symbols) are depicted.

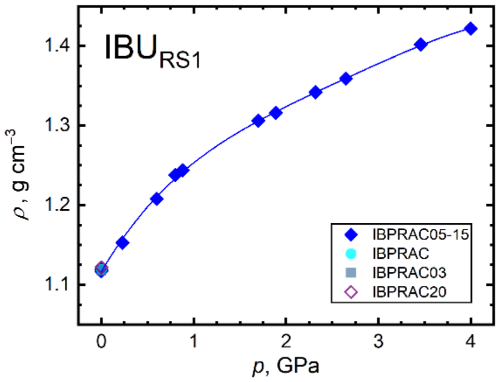

Figure S12. Experimental density data for crystalline ibuprofen at 296 K, all culled from the respective CSD entries. A cubic fit of the variable-pressure density data showing consistency (full symbols) is depicted.

4. Additional results of the quasi-harmonic models

4.1. Components of QHA

Experimental data on heat energy – volume curves $E_{el}(V)$ for polymorphs which are not shown in Figure 3 in the main paper are depicted in Figure S13. Additional characteristics of all the computed $E_{el}(V)$ are given in Table S11.

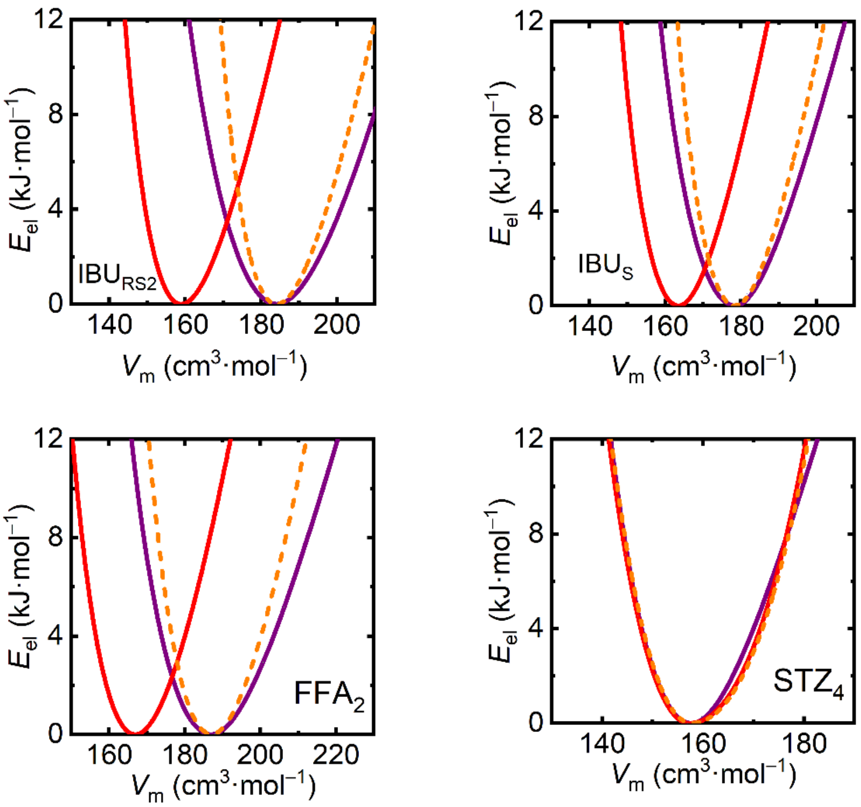

Figure S13. Static electron energy E_{el} as a function of the molar volume of the crystal for selected polymorphs of RS-ibuprofen (IBU_{RS}), S-ibuprofen (IBU_S), flufenamic acid (FFA_2), and sulfathiazole (STZ_4). Data calculated using either DFTB3-D4/3ob (M1, red lines) or PBE-D3(BJ)/PAW (M7, purple) methods. Horizontally shifted DFTB3/3ob curves sharing the minimum point with the PBE-D3(BJ)/PAW curves are depicted with orange dashed lines (M2).

Table S11

Curvatures κ of calculated $E_{el}(V_m)$ curves defined as the second derivatives of the energy with respect to the molar volume, evaluated at the minimum of the $E_{el}(V_m)$ curve. All values given in J mol cm^{-6}.

	Ibuprofen			Flufenamic acid		Sulfathiazole	
	IBU_{RS1}	IBU_{RS2}	IBU_S	FFA_1	FFA_2	STZ_3	STZ_4
M1	88.1	65.4	80.2	75.0	60.3	56.5	34.8
M7	59.6	39.2	59.2	51.4	43.8	79.9	79.1

Table S12

Comparison of experimental unit-cell parameters with their counterparts belonging to optimized unit-cell geometries corresponding to minima of the respective $E_{el}(V_m)$ curves. Raw data given in Å or degrees. Relative percentage deviations of calculated unit-cell parameters δ_i given for reader's convenience. Experimental data entries were collected at 90 to 100 K, always representing reference data relevant at the lowest available temperature. The only exception is the IBU_{RS2} structure investigated at 258 K.

	a	b	c	β	δ_a	δ_b	δ_c
			IBU_{RS1}				
M1	14.144	7.730	9.988	102.01	−2.4%	−1.3%	−4.8%
M7	14.530	7.796	10.368	100.37	0.3%	−0.5%	−1.2%
Exp.[1]	14.485	7.832	10.491	99.74	–	–	–
			IBU_{RS2}				
M1	11.320	4.267	22.410	96.54	−8.6%	−27.3%	27.6%
M7	12.466	5.461	17.654	95.09	0.7%	−7.0%	0.5%
Exp.[2]	12.379	5.872	17.562	94.87	–	–	–
			IBU_S				
M1	11.184	7.837	12.972	109.39	−7.6%	−1.5%	−2.9%
M7	11.929	7.943	13.262	111.25	−1.5%	−0.2%	−0.7%
Exp.[3]	12.109	7.960	13.362	111.95	–	–	–
			FFA_1				
M1	12.150	7.685	12.002	92.78	−2.1%	−0.9%	−5.1%
M7	11.890	7.718	12.568	94.68	−4.2%	−0.5%	−0.6%
Exp.[5]	12.416	7.753	12.647	94.69	–	–	–
			FFA_2				
M1	10.233	10.005	11.633	110.65	−6.0%	−2.3%	−1.0%
M7	10.385	10.236	11.709	111.67	−4.6%	0.0%	−0.3%
Exp.[4]	10.881	10.237	11.749	111.32	–	–	–
			STZ_3				
M1	17.417	8.491	15.495	112.76	12.3%	0.0%	−11.1%
M7	17.450	8.482	15.541	113.33	12.6%	−0.1%	−10.8%
Exp.[17]	15.504	8.494	17.427	112.76	–	–	–
			STZ_4				
M1	10.768	8.213	11.839	86.14	−0.2%	−3.2%	3.9%
M7	10.857	8.472	11.327	90.96	0.6%	−0.1%	−0.6%
Exp.[6]	10.789	8.484	11.398	91.64	–	–	–

Harmonic vibrational frequencies predicted by most DFT functionals are systematically overestimated, which is best illustrated by the blue-shifted positions of the signals around 3000 cm^{-1}, mostly corresponding to the C–H stretch modes in the PBE calculated densities of phonon states in Figure S14. On the other hand, DFTB predicts these signals at lower wavenumbers, being appreciably closer to the real values for organic materials[18] than the PBE frequencies that are commonly overestimated by 4% or more in the high-frequency region.[19-20] Such a better

performance of DFTB for modeling the stretch modes can be traced to its parametrization strategy.[21]

In this context, it is difficult to assess the accuracy of the predicted frequencies of any O–H and N–H stretching modes in our target crystals that vary by hundreds of cm^{-1} upon using either DFTB or PBE methods. Precise positions of the respective phonon signals are strongly affected by the intensity of hydrogen bonding in crystals. Since the enantiopure IBU_S exhibits O–H modes at lower wavenumbers than the racemic IBU_{RS1} phase, one can assume from the PBE phonon frequencies that the hydrogen bonding between the carboxyl groups is stronger in the former structure. Similarly, stretch modes of the O–H donors in flufenamic acid are red-shifted with respect to the stretch modes of the N–H donors. For the larger unit cell of STZ_3, phonon signals are more split in a more complex way than for STZ_4. Hydrogen bonds of the imino N–H_1 donors are predicted to be somewhat stronger in STZ_3, whereas those of the amino N–H_2 donors are stronger in STZ_4.

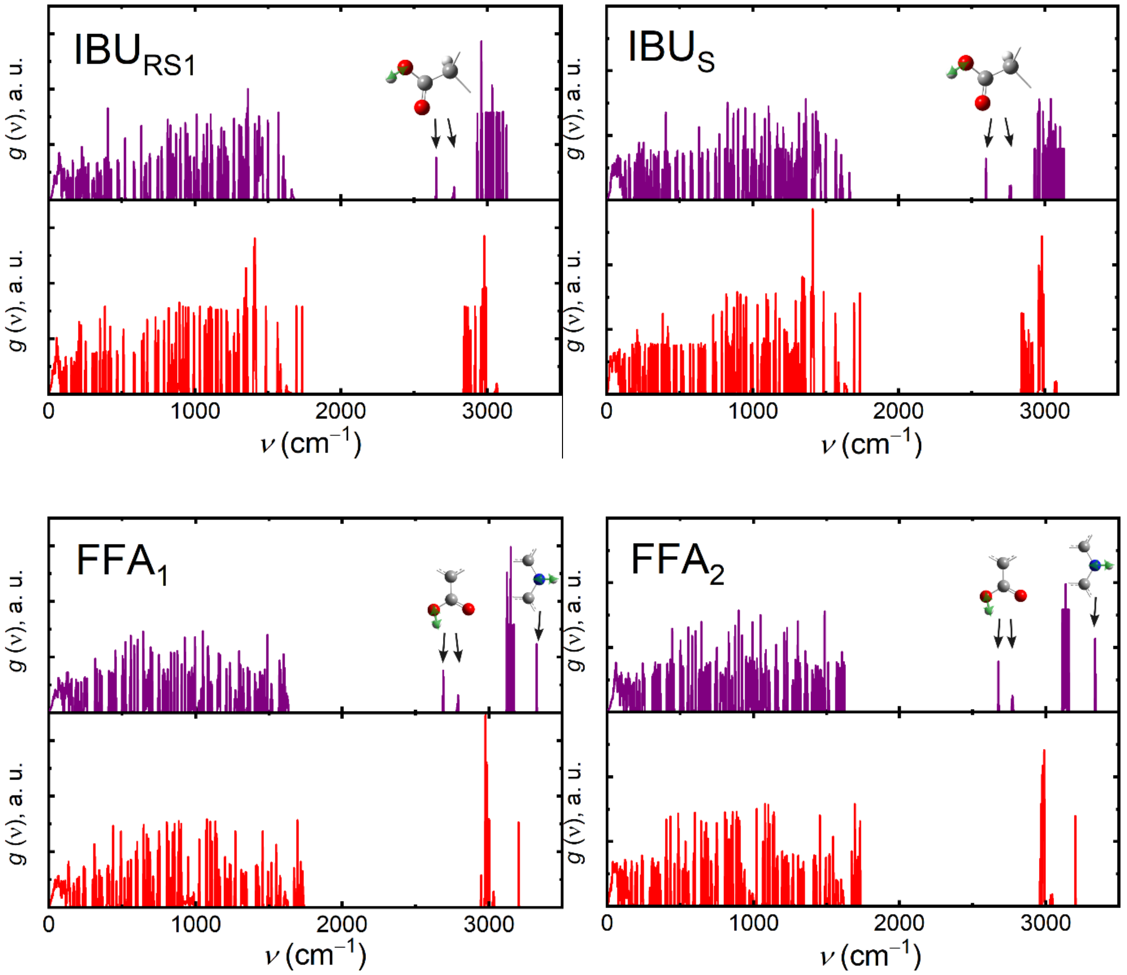

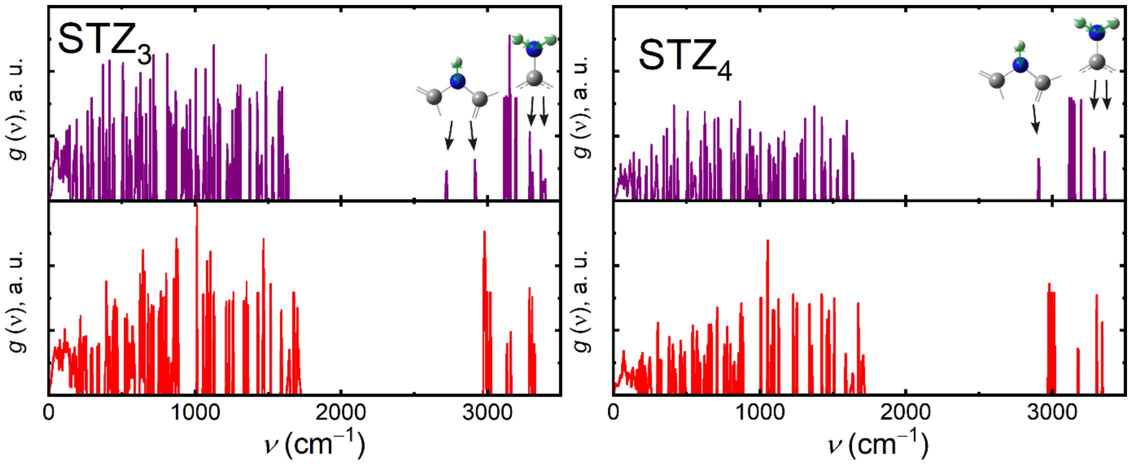

Figure S14. Overview of densities of phonon states calculated for geometries corresponding to the minimum of the $E_{el}(V)$ curve for the target polymorphs of ibuprofen (IBU), flufenamic acid (FFA) and sulfathiazole (STZ). Data in red and purple stand for results of PBE-D3/PAW (M7) and DFTB3-D4/3ob (M1) methods, respectively.

Interestingly, signals of the DFTB density of phonon states signals extend somewhat higher in the lower-frequency region, indicating possibly some gaps in the parametrization strategies not accounting for the valence deformation modes in detail. Unfortunately, in the thermodynamic context, it is the low-frequency region that governs the macroscopic properties of materials at low-to-ambient temperatures. Still, the accurate localization of the stretching modes is important for capturing the zero-point energy contributions to polymorph ranking or sublimation. Densities of phonon states for all considered polymorphs are depicted in Figure S14 and the resulting vibrational Helmholtz energies, which are not shown in Figure 4 in the main paper, are depicted in Figure S15.

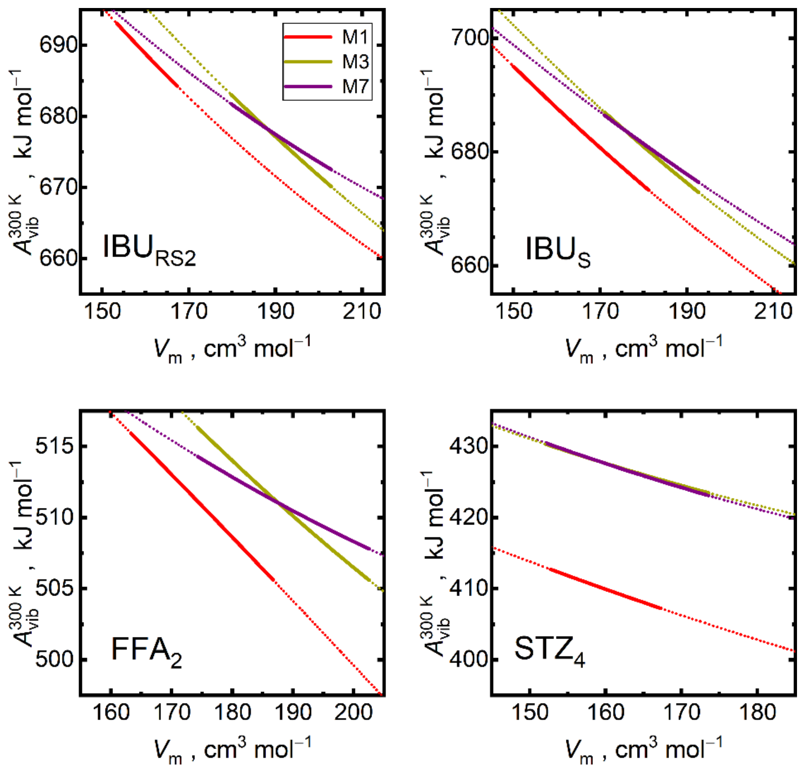

Figure S15. Vibrational Helmholtz energy A_{vib} of target crystals at 300 K as a function of the molar volume for selected polymorphs of ibuprofen (IBU$_{RS2}$, IBU$_S$), flufenamic acid (FFA$_2$), and sulfathiazole (STZ$_4$). Data calculated using either DFTB3/3ob (M1), or PBE-D3(BJ)/PAW (M7) levels of theory, and their M3 composite approach extrapolating single-volume PBE-D3(BJ)/PAW frequencies with DFTB3/3ob Grüneisen parameters (M3). Solid lines represent volume intervals covered explicitly M1 and M7 phonon calculations, whereas dotted lines represent extrapolations of $A_{vib}(V_m)$.

A typical fast decay of the Grüneisen parameters, decreasing from units for the low-frequency lattice modes towards zero for the intramolecular modes can be observed in Figure S16. A detailed look reveals that PBE-D3/PAW yields somewhat lower Grüneisen parameters than DFTB3-D4 for the low-frequency modes. PBE-D3/PAW also captures negative values of the Grüneisen parameters for the O–H and N–H stretching modes, which is a consequence of the hydrogen bonding.[22] The DFTB3-D4 method also yields a larger scatter of results. In this context, attention should be paid to the magnitude of the ΔV_0 off-set. In cases of large ΔV_0, M1 phonon calculations should be performed over a sufficiently broad volume interval that extends to the V_0^{PBE} value to maximize the chances for a sensible phonon extrapolation. In this work, we evaluated the DFTB Grüneisen parameters for each phonon mode from a fit over at least six crystal volumes.

Phonon dispersion was fully accounted for in all considered crystal volumes in the DFTB calculations. Ratio of the Helmholtz vibrational energies obtained with and without the phonon dispersion (including its volume dependence), was then used to scale the Helmholtz energies computed from the Grüneisen-extrapolated PBE frequencies.

Characteristics of the vibrational Helmholtz energies $A_{vib}(V)$ at 300 K with respect to crystal volume are given in Table S13 to enable validation of the newly developed composite M4 model with raw methods M1 and M7.

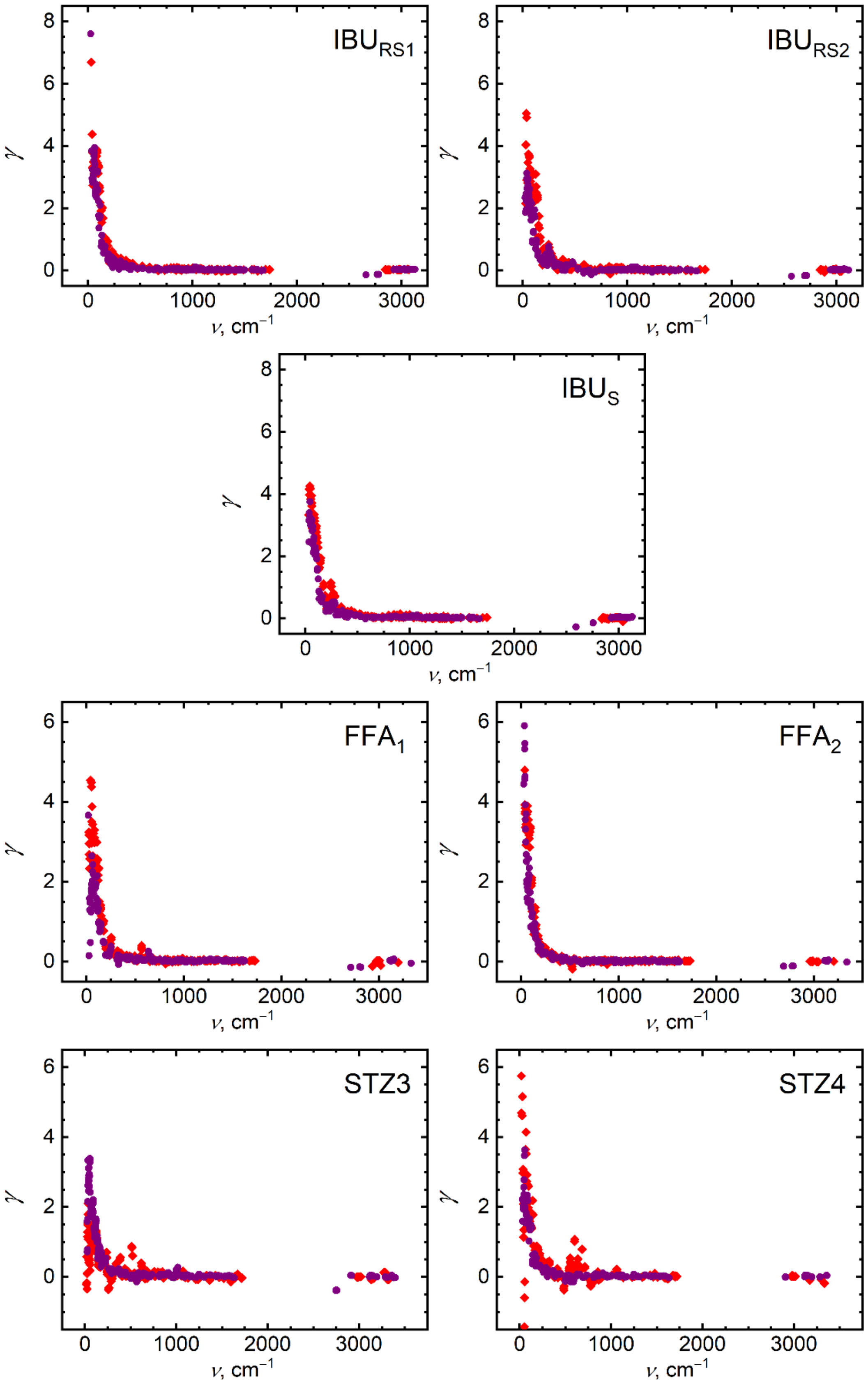

Figure S16. Grüneisen parameters that can be used to extrapolate phonon frequencies of the target molecular crystals. Values determined from fits over phonon frequencies explicitly calculated using PBE-D3/PAW (purple) for 5 crystal volumes and using DFTB3-D4/3ob (red) for at least 6 crystal volumes.

Table S13

Comparison of A_{vib} values (in kJ mol^{-1}) at 300 K, their first derivatives (in J cm^{-3}) and second derivatives (in J mol cm^{-6}) with respect to the molar volume calculated using the raw M1 (DFTB3-D4/3ob) and M7 (PBE-D3/PAW) methods and their composite M4 approach. All data were evaluated for the volumes corresponding to the minima of the respective $E_{el}(V)$ curves.

	M7			M1			M4		
	A_{vib}	$\partial A_{vib}/\partial V_m$	$\partial^2 A_{vib}/\partial V_m^2$	A_{vib}	$\partial A_{vib}/\partial V_m$	$\partial^2 A_{vib}/\partial V_m^2$	A_{vib}	$\partial A_{vib}/\partial V_m$	$\partial^2 A_{vib}/\partial V_m^2$
IBU_{RS1}	682.1	−573.7	4.064	685.5	−738.1	5.079	681.9	−689.5	3.855
IBU_{RS2}	679.9	−423.7	3.301	689.5	−638.9	3.996	680.5	−581.4	2.969
IBU_{S}	682.3	−549.6	2.176	685.2	−704.3	3.244	682.0	−661.7	3.702
FFA_{1}	510.5	−341.2	1.938	513.8	−404.9	2.128	511.2	−369.5	2.647
FFA_{2}	511.2	−233.2	2.098	514.4	−435.4	0.661	511.2	−383.7	2.620
STZ_{3}	427.2	−424.9	6.397	410.1	−357.1	6.409	427.3	−312.0	1.963
STZ_{4}	428.4	−361.4	3.384	411.0	−384.0	2.283	428.4	−335.5	3.245

4.2. Results of QHA

Comparison of the experimental data on molar volumes with the results calculated in this work, which are not shown in Figure 5 in the main document, are depicted in Figure S17. Calculated molar volumes at selected temperatures are given in Table S14. Analysis of the anisotropy of the target crystals is given in Figure S18 and Figure S19. Calculated absolute entropies for all the target crystals at 300 K are listed in Table S15 and their comparison with the pseudo-experimental data is depicted in Figure S20. Analysis of the agreement of individual models with the reference M7 in terms of the first derivatives of Gibbs energy is given in Figure S21. Calculated isobaric heat capacities for all the target crystals at 300 K are listed in Table S16 and their mutual comparison is depicted in Figure S22 (except for IBU_{RS1}, which is shown in Figure 7 in the main text). Calculated coefficients of isobaric thermal expansion for all target materials except IBU_{RS1} (given in Figure 7 in the main text) are depicted in Figure S23. Analysis of the agreement of individual models with the reference M7 in terms of the first derivatives of Gibbs energy is given in Figure S24.

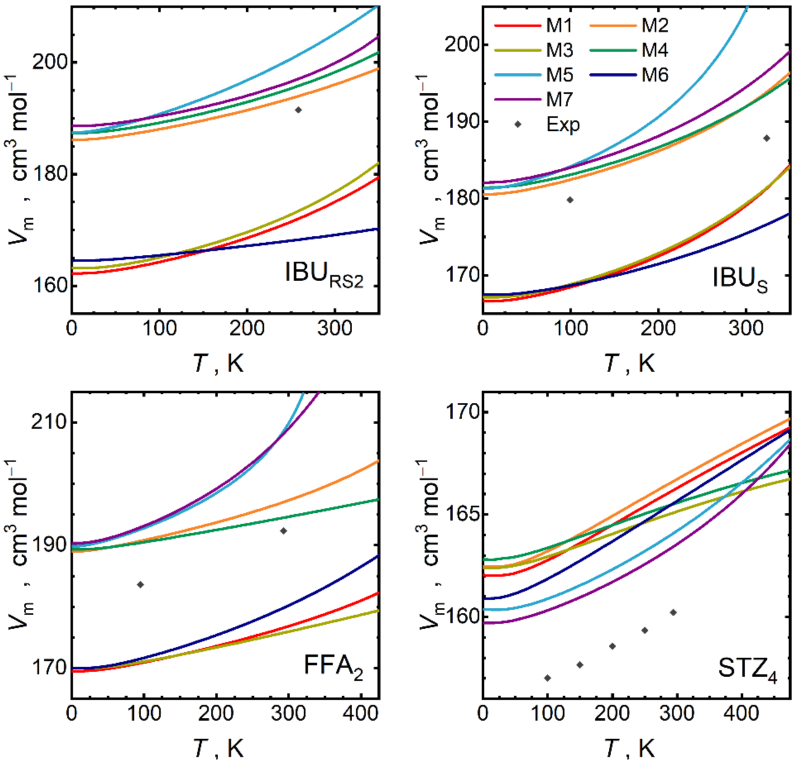

Figure S17. Equilibrium molar volumes for selected target crystal phases at zero pressure calculated using individual formulations of the quasi-harmonic approximation M1 to M7 (colored lines, see Figure 2 in the main paper for explanation), and experimental reference data (gray diamonds).

Table S14

List of equilibrium molar volumes V_m (in cm^3 mol^{-1}) at zero pressure calculated using the raw M1 (DFTB3-D4/3ob) and M7 (PBE-D3/PAW) quasi-harmonic methods and their composite approaches M2–M6.

	M1	M2	M3	M4	M5	M6	M7
				0 K			
IBU_{RS1}	162.2	177.9	162.9	178.8	178.9	163.5	179.6
IBU_{RS2}	162.2	186.1	163.2	187.4	187.5	164.5	188.6
IBU_S	166.6	180.5	167.1	181.4	181.3	167.5	182.1
FFA_1	171.3	187.2	171.7	188.0	188.0	172.8	188.6
FFA_2	169.5	189.0	170.0	189.3	189.8	170.0	190.4
STZ_3	160.9	162.6	160.8	162.6	161.7	161.3	161.9
STZ_4	162.0	162.5	162.4	162.8	160.4	160.9	159.7
				300 K			
IBU_{RS1}	174.7	188.0	176.4	190.0	197.2	173.0	190.0
IBU_{RS2}	175.0	196.1	176.8	198.3	205.2	169.1	199.9
IBU_S	179.1	192.0	179.4	192.0	204.7	175.5	194.5
FFA_1	175.4	191.5	175.6	191.4	195.2	179.5	192.5
FFA_2	176.8	197.3	175.9	194.7	209.9	180.2	209.0
STZ_3	166.2	167.7	166.0	167.7	164.8	169.3	166.4
STZ_4	166.3	166.7	165.2	165.6	164.2	165.7	163.6

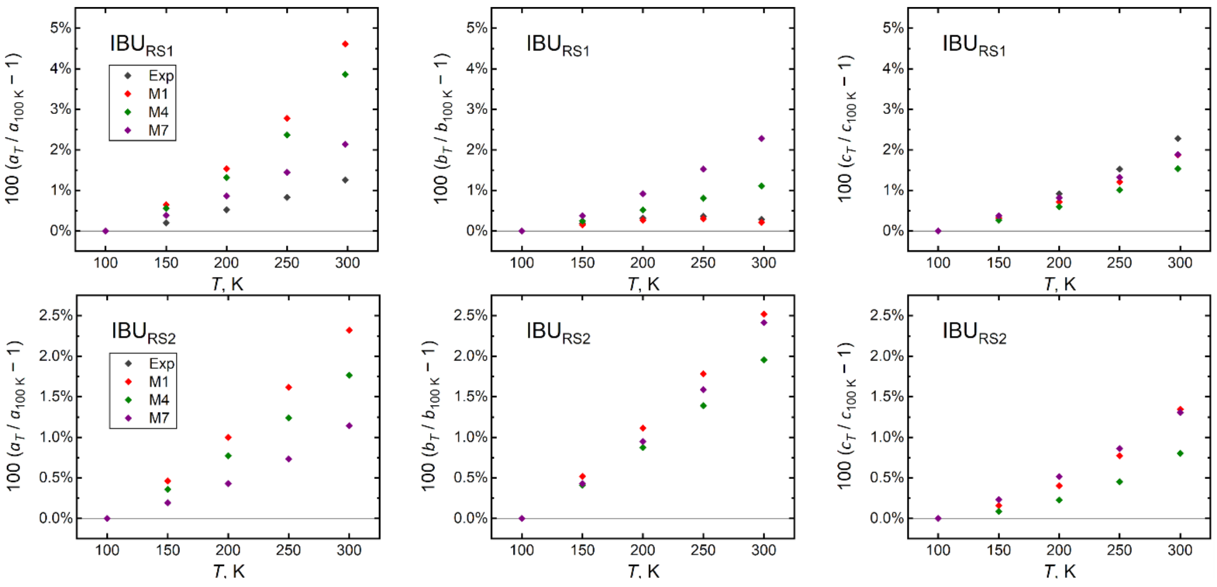

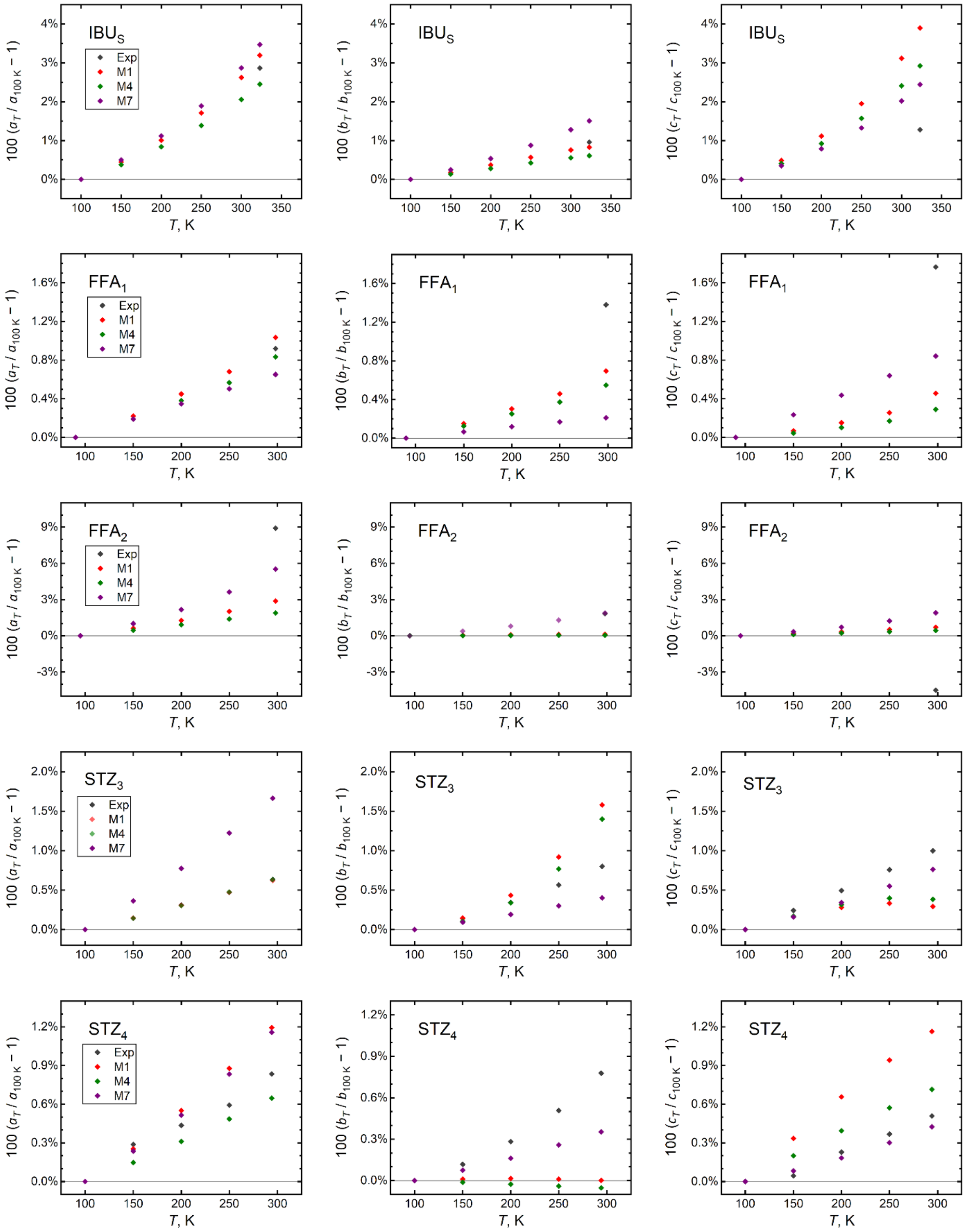

Figure S18. Comparison of thermal expansion of unit cells of target crystal structures in the directions of individual lattice vectors. Data represent relative prolongation of individual unit-cell parameters upon heating from 100 K up to the ambient conditions. Results shown for literature experimental data (sources referenced in Figures S9 to S12) and three selected computational models (M1, M4, M7) adopted in this work.

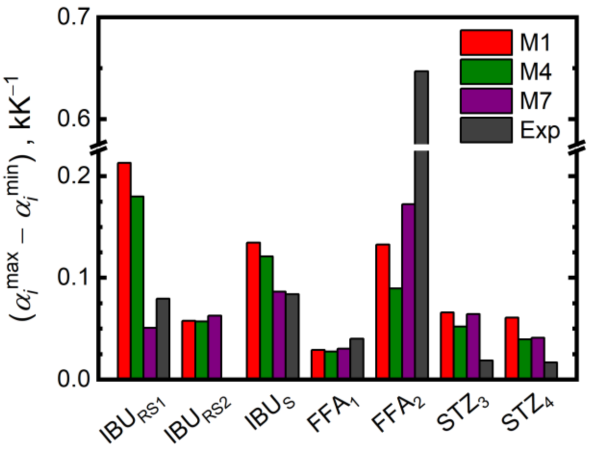

Figure S19. Comparison of thermal expansion of the target crystal structures in terms of the difference of their linear expansion coefficients in the crystallographic directions of minimum and maximum thermal expansion.

Table S15

List of absolute entropies S_m (in J K^{-1} mol^{-1}) at zero pressure calculated using the raw M1 (DFTB3-D4/3ob) and M7 (PBE-D3/PAW) quasi-harmonic methods and their composite approaches M2–M6 and pseudo-experimental values (EXP).

	M1	M2	M3	M4	M5	M6	M7	EXP
				300 K				
IBU_{RS1}	336.0	360.8	312.9	337.7	376.9	307.0	331.8	333.7
IBU_{RS2}	319.8	353.0	302.7	336.0	366.1	305.2	334.3	n/a
IBU_S	336.9	361.9	313.2	335.7	386.5	306.2	333.7	338.0
FFA_1	313.7	327.2	314.1	325.0	330.4	314.2	326.7	344.1
FFA_2	315.7	340.5	303.2	320.6	356.9	297.8	340.9	n/a
STZ_3	300.3	301.5	275.9	277.2	299.2	280.8	277.9	277.9
STZ_4	298.4	298.8	267.8	268.0	296.6	272.7	270.6	270.4

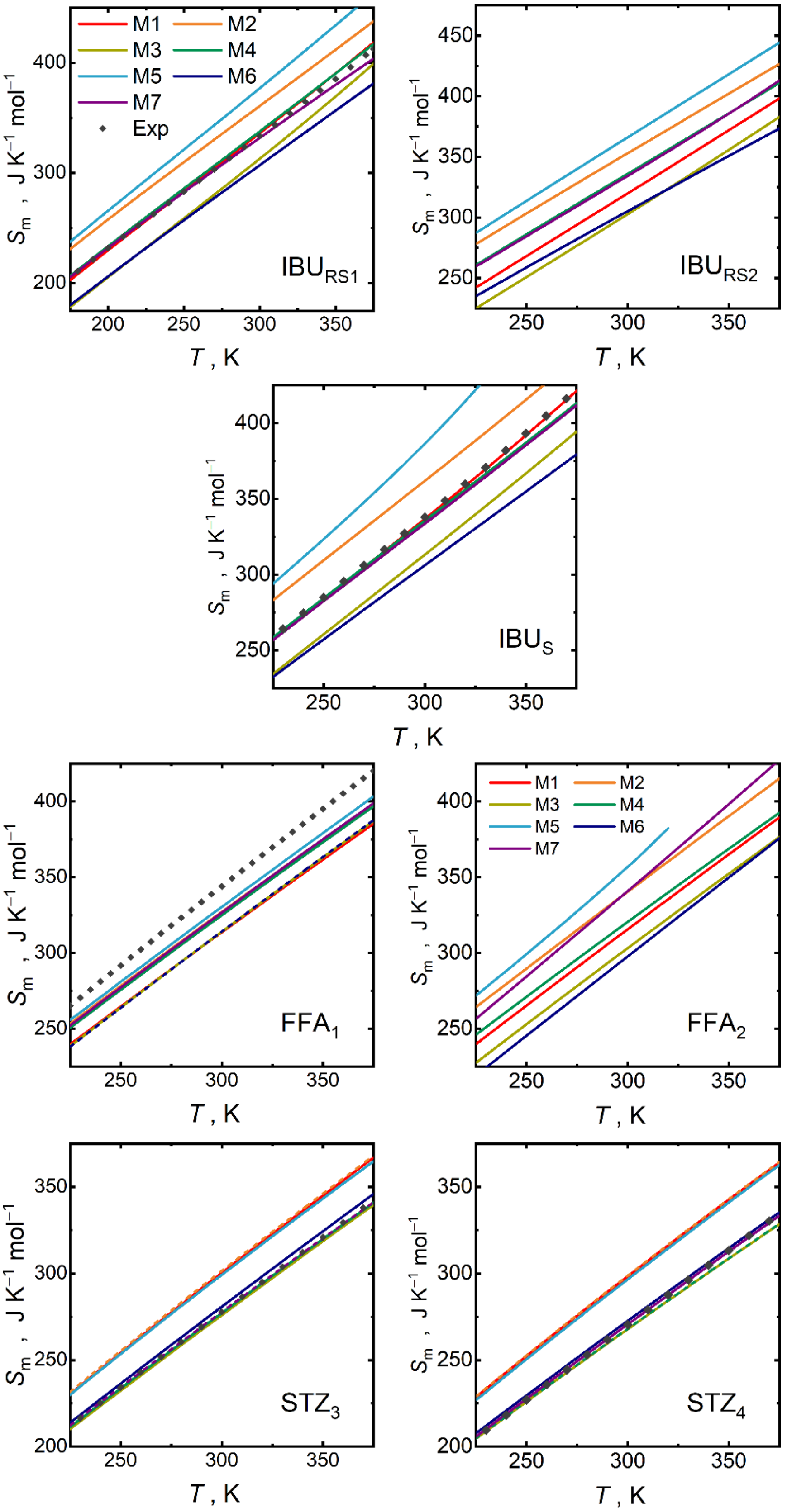

Figure S20. Absolute entropies of selected target crystal phases at zero pressure calculated using individual formulations of the quasi-harmonic approximation M1 to M7 (colored lines, see Figure 2 in the main paper for explanation), and pseudo-experimental reference data (gray diamonds). Dashed lines are optionally used to make multiple overlapping curves visible.

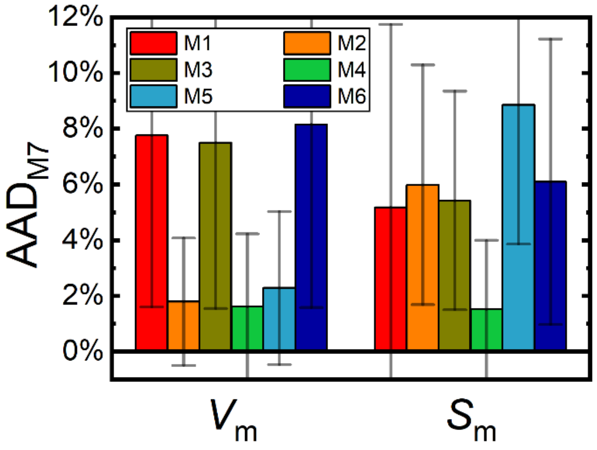

Figure S21. Absolute average deviations (AAD) of molar volumes (V_m) and absolute entropies (S_m) at 300 K and zero pressure calculated using individual formulations of the quasi-harmonic approximation (M1 to M6, see **Chyba! Nenalezen zdroj odkazů.** for explanation) from the results of the M7 model. Standard deviations of the errors of calculated properties that were observed for individual target crystal structures are depicted as error bars to illustrate scatter of computational errors yielded by the considered methods.

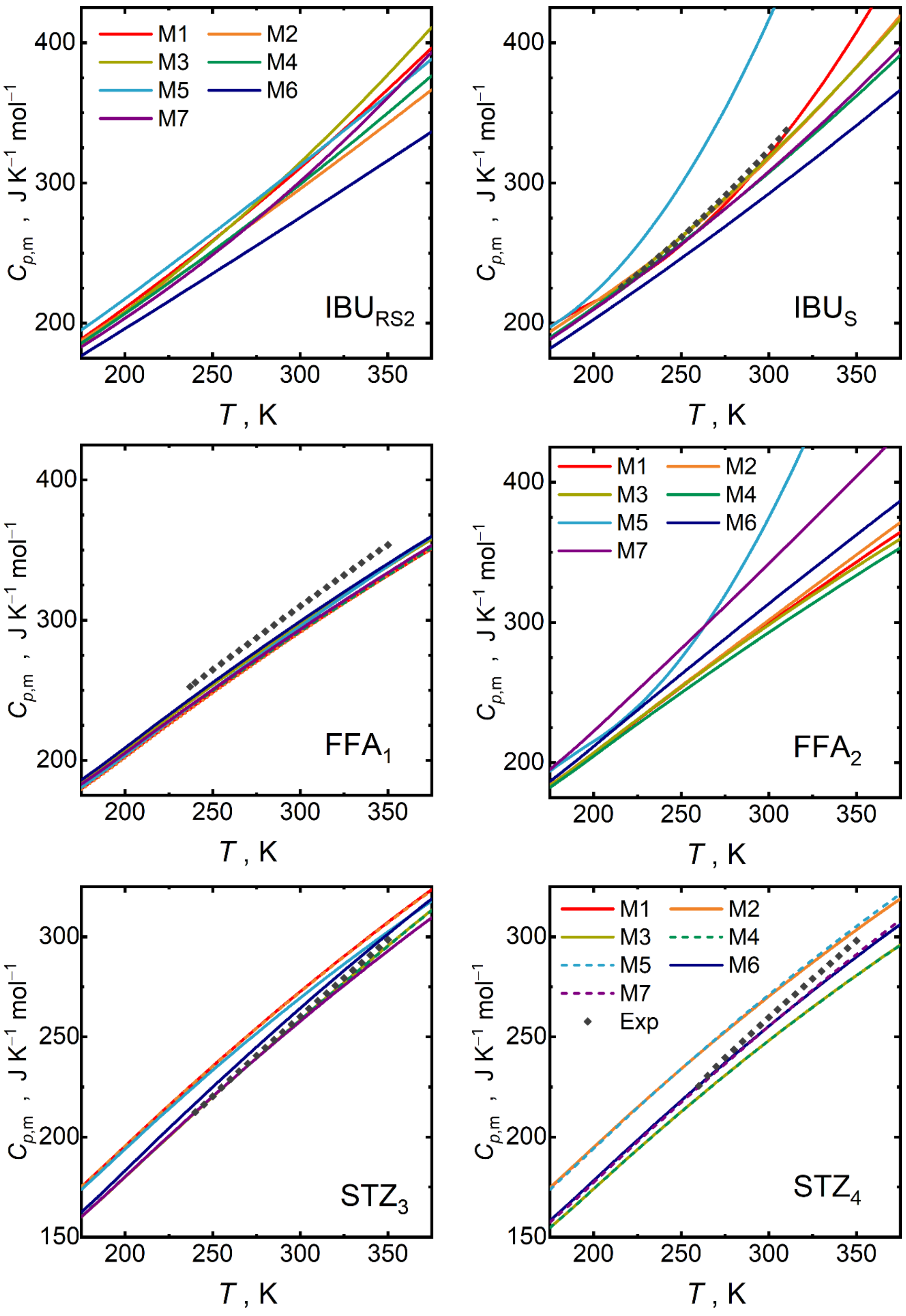

Figure S22. Isobaric heat capacities of selected target crystal phases at zero pressure calculated using individual formulations of the quasi-harmonic approximation M1 to M7 (colored lines, see Figure 2 in the main paper for explanation), and experimental reference data (gray diamonds). Dashed lines are optionally used to make multiple overlapping curves visible.

Table S16

List of isobaric heat capacities $C_{p,\mathrm{m}}$ (in J K^{-1} mol^{-1}) at zero pressure calculated using the raw M1 (DFTB3-D4/3ob) and M7 (PBE-D3/PAW) quasi-harmonic methods and their composite approaches M2–M6 and the respective experimental values.

	M1	M2	M3	M4	M5	M6	M7	EXP
				300 K				
IBU_{RS1}	321.7	305.7	332.1	313.0	336.2	297.5	291.1	305.3
IBU_{RS2}	310.7	295.7	314.5	299.0	312.5	275.3	301.0	n/a
IBU_{S}	320.0	317.3	318.7	307.3	416.5	292.3	308.2	323.5
FFA_{1}	291.6	291.1	297.3	292.1	295.6	299.2	293.4	310.0
FFA_{2}	299.3	301.7	298.3	292.9	374.7	313.3	341.9	n/a
STZ_{3}	272.7	272.5	258.5	258.5	269.5	264.4	257.8	260.2
STZ_{4}	270.3	270.2	248.2	248.1	271.1	255.3	255.3	259.7

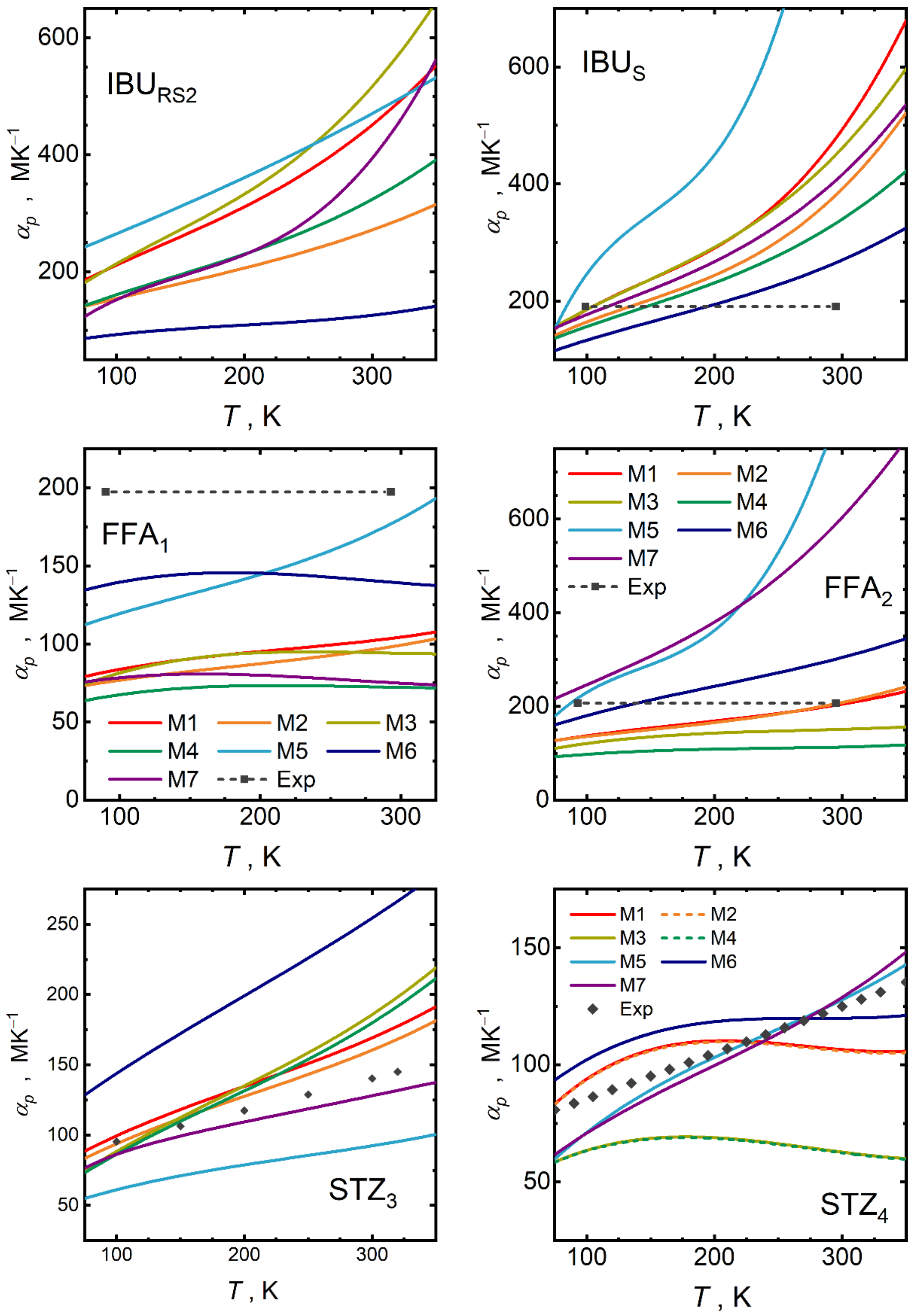

Figure S23. Isobaric thermal expansivity coefficients of selected target crystal phases at zero pressure calculated using individual formulations of the quasi-harmonic approximation M1 to M7 (colored lines, see Figure 2 in the main paper for explanation), and experimental reference data (gray diamonds – rigorously derived from variable-temperature experimental data for STZ_3 and STZ_4; gray dashed lines – tentative estimate from two literature data points). Colored dashed lines are optionally used to make multiple overlapping curves visible.

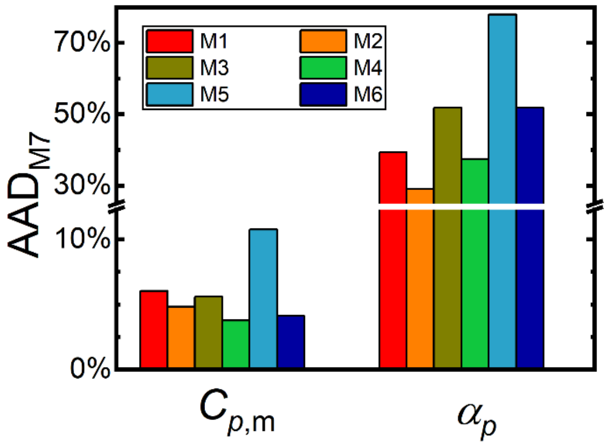

Figure S24. Absolute average deviations (AAD) of isobaric molar heat capacities ($C_{p,\mathrm{m}}$) and isobaric thermal expansion coefficients (α_p) at 300 K and zero pressure calculated using individual formulations of the quasi-harmonic approximation (M1 to M7, see Figure 2 for explanation) from the results of the M7 model.

5. Pair interactions and polymorph ranking for crystalline API

Results of the fragment-based ab initio refinement of the lattice energy of all three ranked polymorph pairs are provided in this section. Figure S25 depicts the complete results on polymorph ranking modeled in this work. Pair interaction energies within a cut-off 8 Å are calculated with DFTB3-D4/3ob, PBE-D3(BJ)/PAW, and MP2C-F12/aug-cc-pVDZ levels of theory in order to refine the polymorph ranking. Differences between the pair interactions modeled by these methods are also given for comparison. While all methods provide comparable pair interactions for the proximate dimers of ibuprofen and flufenamic acid, DFTB fails to reproduce the ab initio interaction energies of the closest dimers of sulfathiazole. Data for ibuprofen, flufenamic acid, and sulfathiazole are given in Figure S26, Figure S27, and Figure S28, respectively, along with the dimer geometries exhibiting the most significant interactions in the crystal lattice. Final ab initio corrections to the monomer and dimer energies of all polymorphs are listed in Table S17. Results of a sensitivity analysis of the ΔG curves are presented in Figure S29 and Figure S30.

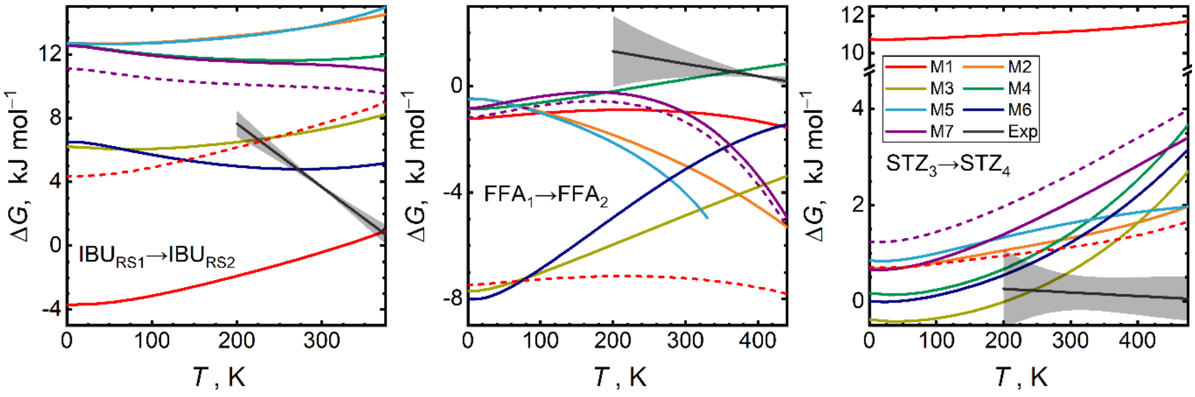

Figure S25. Gibbs energy change ΔG related to the phase transition between the target polymorphs of RS-ibuprofen, flufenamic acid and sulfathiazole. Experimental data and their expanded uncertainties are depicted with gray lines and shaded areas, respectively. Single-point MP2C-F12/aug-cc-pVDZ refinement of proximate pair interaction energies within a 8 Å cut-off distance in the crystal lattice and a MP2/aug-cc-pVDZ refinement of the monomer energies were applied in all cases of the solid colored lines (with respect to DFTB3 energies for M1, and with respect to PBE elsewhere). Results of raw PBE-D3/PAW and DFTB3-D4/3ob electronic energies without this refinement are given with dashed colored lines.

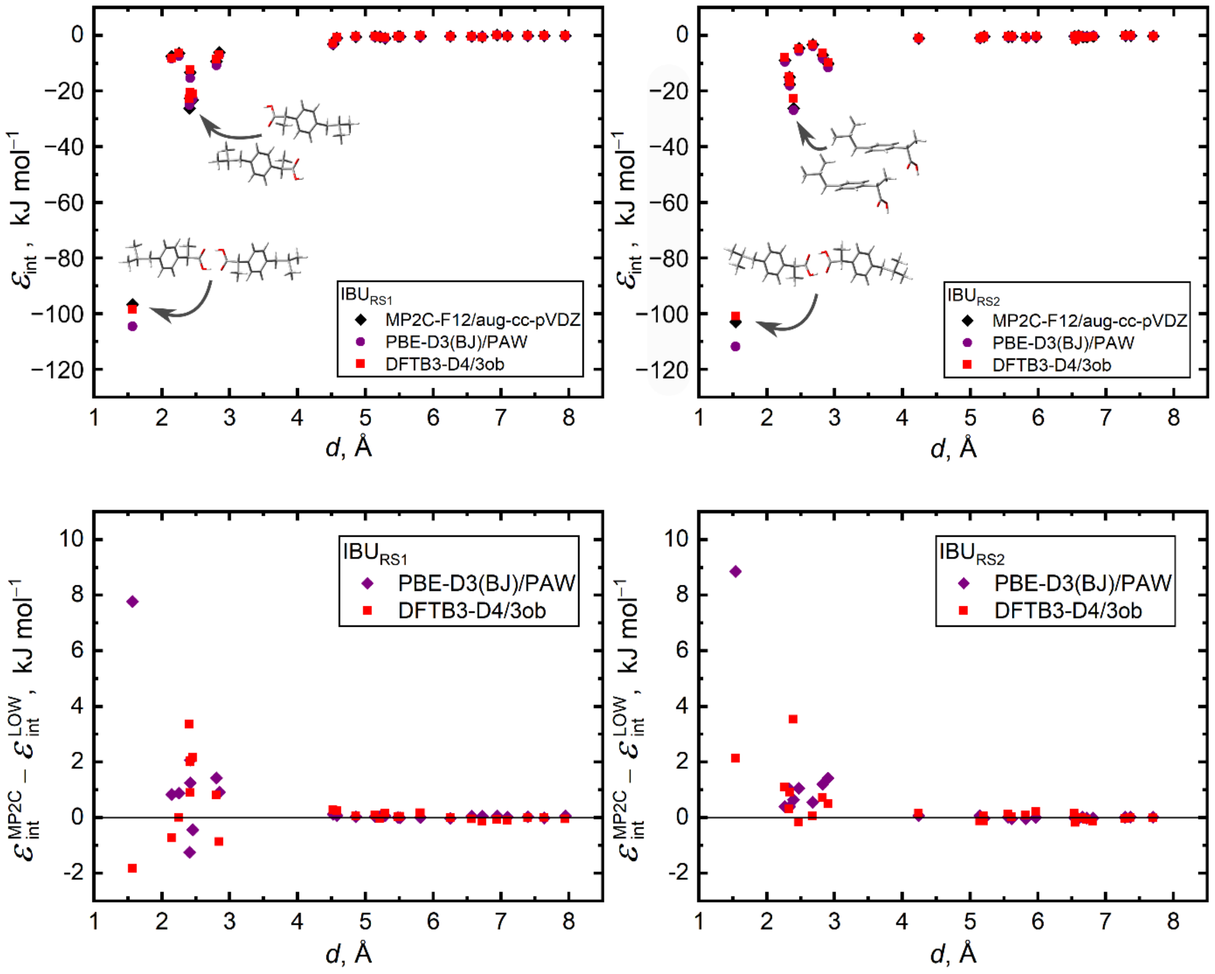

Figure S26. Pair interactions calculated for proximate molecular pairs within the crystal lattices of two polymorphs of racemic ibuprofen using three quantum-chemical levels of theory. Data shown with respect to the distance *d* related to the closest atomic contact of the two molecules within a dimer.

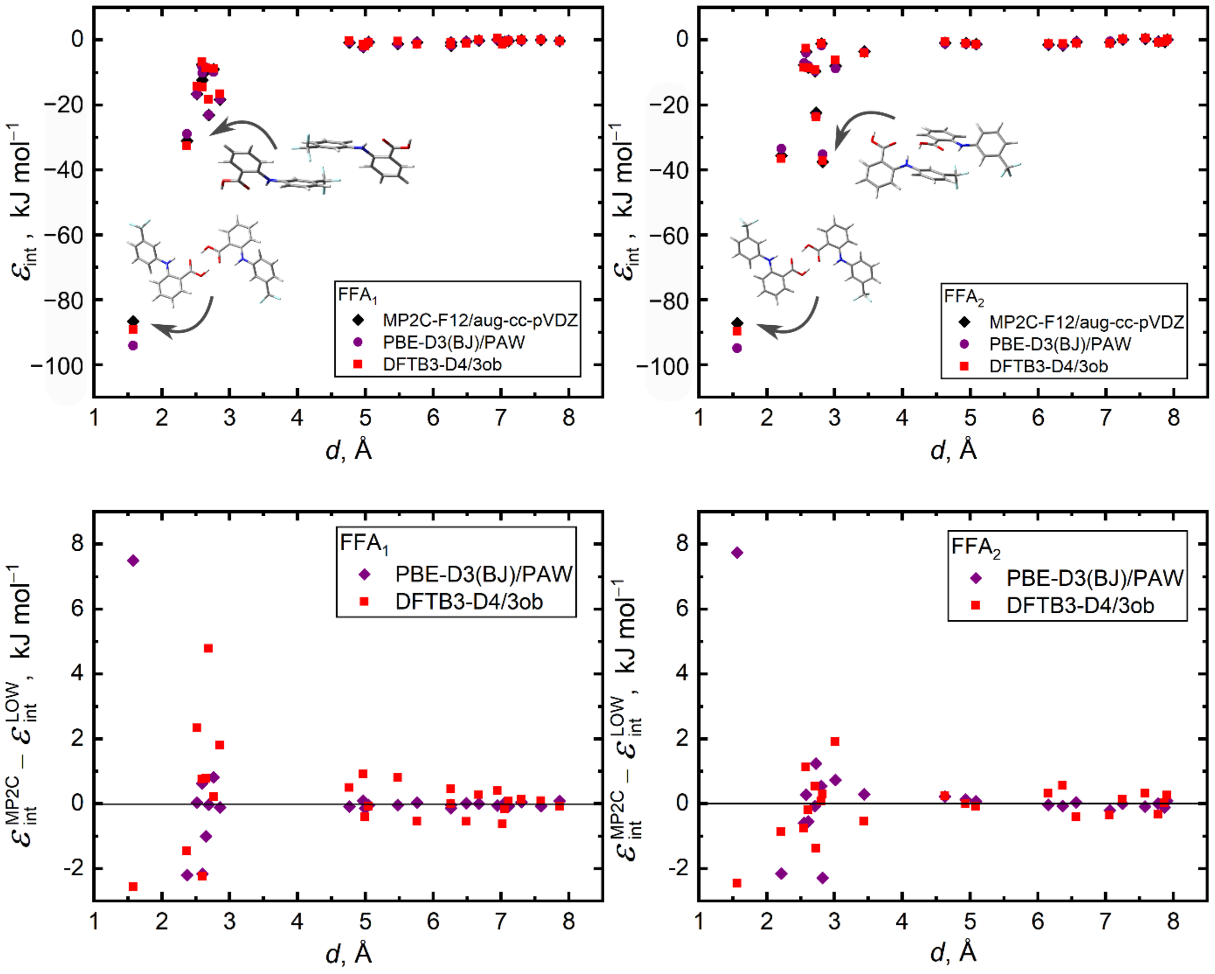

Figure S27. Pair interactions calculated for proximate molecular pairs within the crystal lattices of two polymorphs of flufenamic acid using three quantum-chemical levels of theory. Data shown with respect to the distance *d* related to the closest atomic contact of the two molecules within a dimer.

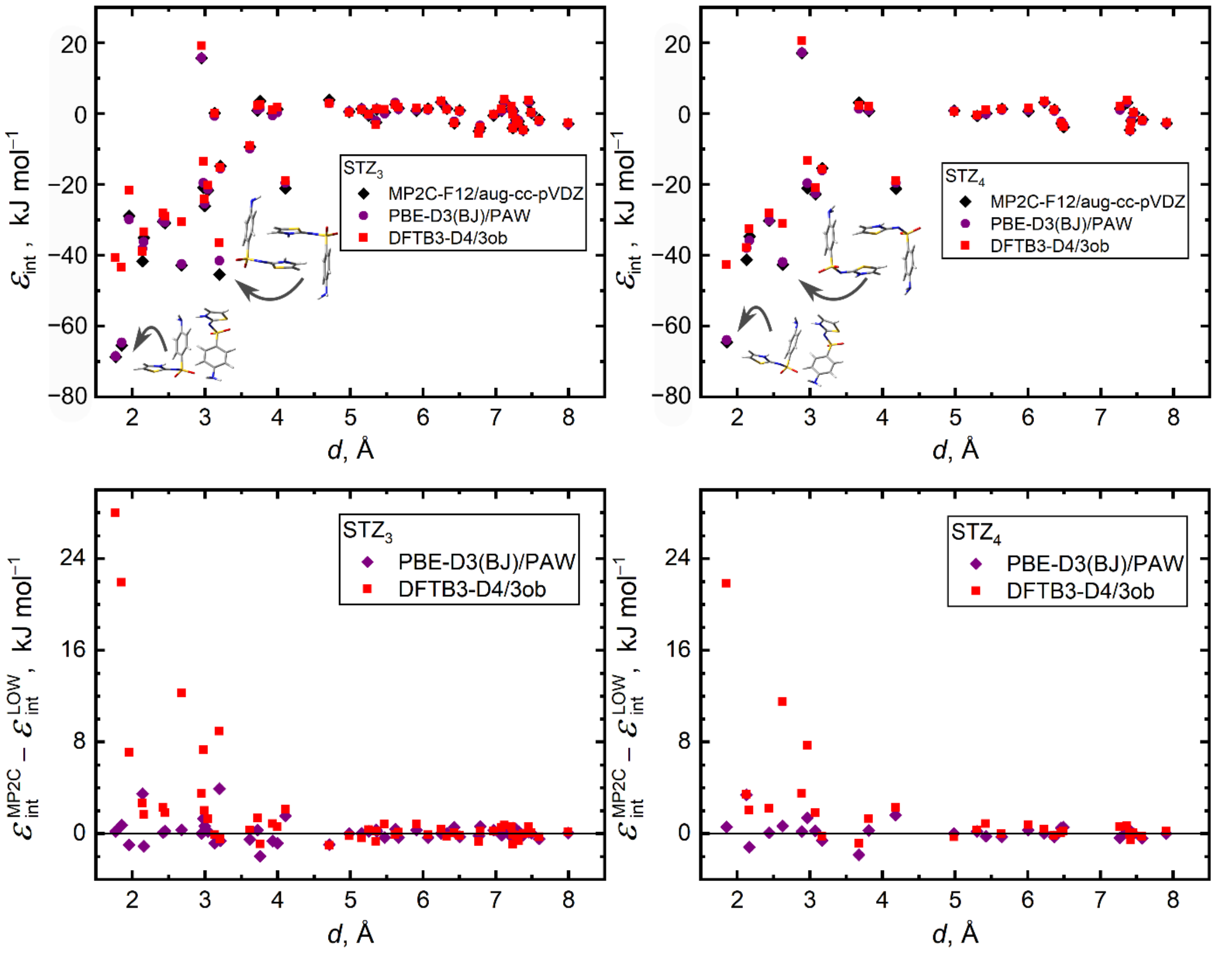

Figure S28. Pair interactions calculated for proximate molecular pairs within the crystal lattices of two polymorphs of sulfathiazole using three quantum-chemical levels of theory. Data shown with respect to the distance d related to the closest atomic contact of the two molecules within a dimer.

Table S17

Ab initio corrections to the energies of target crystals (all related to monomers, in kJ mol^{-1}). M1 (DFTB3-D4/3ob) and M7 (PBE-D3/PAW) quasi-harmonic methods and their composite approaches M2–M6.

	IBU_{RS1}	
Low tier method	DFTB3	PBE
MP2/avdz monomer correction	6.52	−0.48
MP2C/avdz dimer correction	−4.77	8.47
Total correction	1.75	7.99
	IBU_{RS2}	
Low tier method	DFTB3	PBE
MP2/avdz monomer correction	0.00	0.00
MP2C/avdz dimer correction	−6.31	9.42
Total correction	−6.31	9.42
	FFA_1	
Low tier method	DFTB3	PBE
MP2/avdz monomer correction	−0.34	0.54
MP2C/avdz dimer correction	−6.53	1.14
Total correction	−6.87	1.68
	FFA_2	
Low tier method	DFTB3	PBE
MP2/avdz monomer correction	0.00	0.00
MP2C/avdz dimer correction	−0.61	2.02
Total correction	−0.61	2.02
	STZ_3	
Low tier method	DFTB3	PBE
MP2/avdz monomer correction	−3.25	0.52
MP2C/avdz dimer correction	−50.07	−2.09
Total correction	−53.31	−1.56
	STZ_4	
Low tier method	DFTB3	PBE
MP2/avdz monomer correction	0.00	0.00
MP2C/avdz dimer correction	−43.28	−2.14
Total correction	−43.28	−2.14

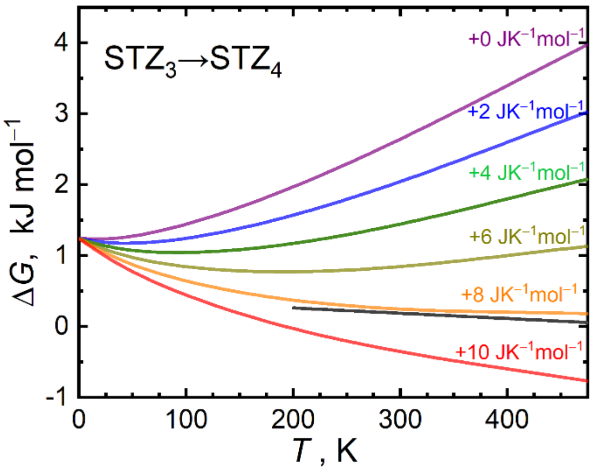

Figure S29. Sensitivity analysis of the Gibbs energy change ΔG related to the phase transition between the target polymorphs of sulfathiazole. Experimental data are depicted with gray line. Colored lines represent results of raw PBE-D3/PAW quasi-harmonic ranking where the absolute molar entropy of phase STZ_4 was artificially increased by given constant values.

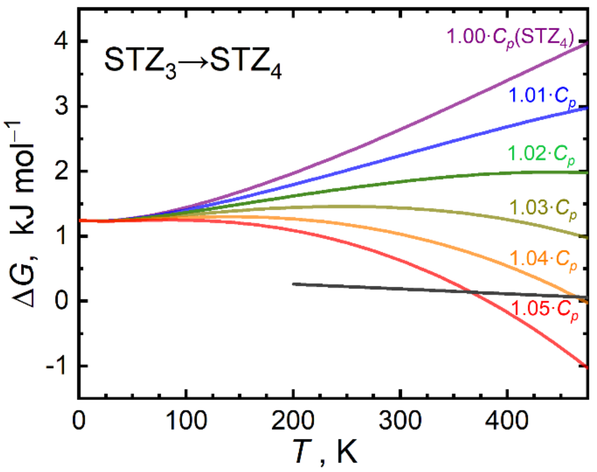

Figure S30. Sensitivity analysis of the Gibbs energy change ΔG related to the phase transition between the target polymorphs of sulfathiazole. Experimental data are depicted with gray line. Colored lines represent results of raw PBE-D3/PAW quasi-harmonic ranking where the isobaric heat capacity of phase STZ_4 was artificially scaled by given values.

6. References to the SI

1. Ostrowska, K.; Kropidłowska, M.; Katrusiak, A., High-Pressure Crystallization and Structural Transformations in Compressed R,S-Ibuprofen. *Crystal Growth & Design* **2015,** *15* (3), 1512-1517.
2. Derollez, P.; Dudognon, E.; Affouard, F.; Danede, F.; Correia, N. T.; Descamps, M., Ab initio structure determination of phase II of racemic ibuprofen by X-ray powder diffraction. *Acta Crystallographica Section B* **2010,** *66* (1), 76-80.
3. King, M. D.; Buchanan, W. D.; Korter, T. M., Understanding the Terahertz Spectra of Crystalline Pharmaceuticals: Terahertz Spectroscopy and Solid-State Density Functional Theory Study of (S)-(+)-Ibuprofen and (RS)-Ibuprofen. *Journal of Pharmaceutical Sciences* **2011,** *100* (3), 1116-1129.
4. López-Mejías, V.; Kampf, J. W.; Matzger, A. J., Nonamorphism in Flufenamic Acid and a New Record for a Polymorphic Compound with Solved Structures. *Journal of the American Chemical Society* **2012,** *134* (24), 9872-9875.
5. Delaney, S. P.; Smith, T. M.; Korter, T. M., Conformational origins of polymorphism in two forms of flufenamic acid. *Journal of Molecular Structure* **2014,** *1078*, 83-89.
6. Sovago, I.; Gutmann, M. J.; Hill, J. G.; Senn, H. M.; Thomas, L. H.; Wilson, C. C.; Farrugia, L. J., Experimental Electron Density and Neutron Diffraction Studies on the Polymorphs of Sulfathiazole. *Crystal Growth & Design* **2014,** *14* (3), 1227-1239.
7. Hourahine, B.; Aradi, B.; Blum, V.; Bonafé, F.; Buccheri, A.; Camacho, C.; Cevallos, C.; Deshaye, M. Y.; Dumitrică, T.; Dominguez, A.; Ehlert, S.; Elstner, M.; van der Heide, T.; Hermann, J.; Irle, S.; Kranz, J. J.; Köhler, C.; Kowalczyk, T.; Kubař, T.; Lee, I. S.; Lutsker, V.; Maurer, R. J.; Min, S. K.; Mitchell, I.; Negre, C.; Niehaus, T. A.; Niklasson, A. M. N.; Page, A. J.; Pecchia, A.; Penazzi, G.; Persson, M. P.; Řezáč, J.; Sánchez, C. G.; Sternberg, M.; Stöhr, M.; Stuckenberg, F.; Tkatchenko, A.; Yu, V. W.-z.; Frauenheim, T., DFTB+, a software package for efficient approximate density functional theory based atomistic simulations. *The Journal of Chemical Physics* **2020,** *152* (12).
8. Štejfa, V.; Rohlíček, J.; Červinka, C., Phase behaviour and heat capacities of selected 1-ethyl-3-methylimidazolium-based ionic liquids II. *J. Chem. Thermodyn.* **2021,** *160*, 106392.
9. Höhne, G. W. H.; Hemminger, W. F.; Flammersheim, H.-J., *Differential Scanning Calorimetry*. 2 ed.; Springer Verlag: Berlin, 2003.
10. Štejfa, V.; Vojtíšková, O.; Pokorný, V.; Rohlíček, J.; Růžička, K.; Fulem, M., Heat capacities of nifedipine, griseofulvin, probucol, and 5,5-diphenylhydantoin. *J. Therm. Anal. Calorim.* **submitted**.
11. Kuhnert-Brandstätter, M.; Borka, L.; Friedrich-Sander, G., Zur Polymorphie von Arzneimitteln: Flufenaminsäure und BL 191. *Arch. Pharm.* **1974,** *307* (11), 845-853.
12. Surov, A. O.; Terekhova, I. V.; Bauer-Brandl, A.; Perlovich, G. L., Thermodynamic and Structural Aspects of Some Fenamate Molecular Crystals. *Crystal Growth & Design* **2009,** *9* (7), 3265-3272.
13. Anwar, J.; Tarling, S. E.; Barnes, P., Polymorphism of sulfathiazole. *J. Pharm. Sci.* **1989,** *78* (4), 337-342.
14. Clout, A.; Buanz, A. B. M.; Prior, T. J.; Reinhard, C.; Wu, Y.; O'Hare, D.; Williams, G. R.; Gaisford, S., Simultaneous Differential Scanning Calorimetry-Synchrotron X-ray Powder Diffraction: A Powerful Technique for Physical Form Characterization in Pharmaceutical Materials. *Anal. Chem.* **2016,** *88* (20), 10111-10117.
15. Abu Bakar, M. R.; Nagy, Z. K.; Rielly, C. D.; Dann, S. E., Investigation of the riddle of sulfathiazole polymorphism. *International Journal of Pharmaceutics* **2011,** *414* (1), 86-103.

16. Štejfa, V.; Pokorný, V.; Mathers, A.; Růžička, K.; Fulem, M., Heat capacities of selected active pharmaceutical ingredients. *The Journal of Chemical Thermodynamics* **2021,** *163*, 106585.
17. Drebushchak, T. N.; Boldyreva, E. V.; Mikhailenko, M. A., Experimental Crystal Structure Determination. *Zhurnal Strukturnoi Khimii* **2008,** *49*, 90.
18. Schimanouchi, T., *Tables of Molecular Vibrational Frequencies Consolidated Volume I*. National Bureau of Standards: Washington, D.C., 1972.
19. Červinka, C.; Fulem, M.; Růžička, K., Evaluation of Accuracy of Ideal-Gas Heat Capacity and Entropy Calculations by Density Functional Theory (DFT) for Rigid Molecules. *Journal of Chemical and Engineering Data* **2012,** *57* (1), 227-232.
20. Merrick, J. P.; Moran, D.; Radom, L., An Evaluation of Harmonic Vibrational Frequency Scale Factors. *The Journal of Physical Chemistry A* **2007,** *111* (45), 11683-11700.
21. Gaus, M.; Goez, A.; Elstner, M., Parametrization and Benchmark of DFTB3 for Organic Molecules. *Journal of Chemical Theory and Computation* **2013,** *9* (1), 338-354.
22. Červinka, C.; Fulem, M.; Stoffel, R. P.; Dronskowski, R., Thermodynamic properties of molecular crystals calculated within the quasi-harmonic approximation. *J. Phys. Chem. A* **2016,** *120*, 2022-2034.
